# Self-Organized Explainable Symbolic EEG Feature Engineering Using Directed Lobish and Previous-Next Pattern: A Proof-of-Concept Study on Dementia Classification

**DOI:** 10.3390/jcm15218572

**Published:** 2026-11-03

**Authors:** Ferhat Balgetir, Prabal Datta Barua, Jun Shen, Padmanesan Narasimhan, Smitha Shivshankar, Sengul Dogan, Irem Tasci, Burak Tasci, Mehmet Baygin, Turker Tuncer, Makiko Kobayashi

**Affiliations:** 1 Department of Neurology, School of Medicine, Firat University, 23119 Elazig, Türkiye; ferhatbalgetir@firat.edu.tr (F.B.); itasci@firat.edu.tr (I.T.); 2 Faculty of Engineering and Information Technology, University of Technology Sydney, Sydney, NSW 2007, Australia; prabal.barua@usq.edu.au; 3 Australian International Institute of Higher Education, Brisbane, QLD 4000, Australia; smitha.shivshankar@aiihe.edu.au; 4 Department of Computer Engineering, College of Engineering, Erzurum Technical University, 25050 Erzurum, Türkiye; 5 School of Mathematics, Physics and Computing, University of Southern Queensland, Toowoomba, QLD 4350, Australia; 6 School of Computing and Information Technology, University of Wollongong, Wollongong, NSW 2522, Australia; jshen@uow.edu.au; 7 School of Population Health, UNSW Sydney, Kensington, NSW 2052, Australia; 8 Department of Digital Forensics Engineering, Technology Faculty, Firat University, 23119 Elazig, Türkiye; turkertuncer@firat.edu.tr; 9 Vocational School of Technical Sciences, Firat University, 23119 Elazig, Türkiye; 10 Faculty of Advanced Science and Technology, Kumamoto University, Kumamoto 860-8555, Japan

**Keywords:** PNPat, explainable feature engineering, Directed Lobish, combinational majority voting, EEG signal classification

## Abstract

**Objective**: Dementia is a prevalent neurological disorder, and accurate detection is essential for timely intervention. This study aims to design an interpretable symbolic feature engineering framework for EEG-based dementia classification and to examine how its performance depends on the validation protocol. **Methods**: We used a publicly available EEG dementia dataset including Alzheimer’s disease (AD), frontotemporal dementia (FD), and healthy controls. A self-organized explainable feature engineering (SOXFE) model was introduced with five phases: (i) feature extraction using Bipartite Channel Transformer (BCT) and Previous-Next Pattern (PNPat), (ii) feature selection via cumulative weight-based iterative neighborhood component analysis (CWINCA), (iii) explainable output generation using Directed Lobish (DLob), (iv) classification through an ensemble tkNN classifier, and (v) information fusion with combinational majority voting (CMV). Performance was evaluated with segment-wise 10-fold cross-validation, leave-one-subject-out (LOSO) cross-validation, and nested participant-grouped 10-fold cross-validation. **Results**: Segment-wise 10-fold cross-validation, which allowed segments of one participant in both training and test folds, yielded accuracies of 99.12–99.74%. Participant-independent accuracy was substantially lower: 51.19–63.81% for LOSO and 49.00–62.50% for nested participant-grouped validation at the segment level. Participant-level LOSO accuracy equaled the majority-class rate for dementia versus healthy controls (67.05%) and fell below it for AD versus FD (57.63% versus 61.02%). No exact McNemar comparison showed an accuracy advantage over the majority-class baseline. DLob outputs were produced as feature-level visualizations. **Conclusions**: The findings support the feasibility of interpretable, algorithm-derived symbolic feature representations. They do not establish reproducible disease-related EEG characteristics or clinical diagnostic utility. DLob outputs describe the channel transitions used by the model. Independent cohorts and stronger participant-independent performance are required before clinical application.

## 1. Introduction

Dementia is a progressive neurological disorder that significantly impacts cognitive abilities such as memory, decision-making, and communication [1]. The global prevalence of dementia necessitates advanced diagnostic tools that detect the condition and provide interpretable insights into its underlying mechanisms [2]. Electroencephalography (EEG), a noninvasive technique, has become a valuable resource for identifying dementia-related patterns and offers the potential for improved diagnostic accuracy [3].

Machine learning and explainable artificial intelligence (XAI) have been applied to dementia assessment [2,4]. These methods can make data-driven decisions more transparent [5]. Explanations must also be evaluated against independent evidence before clinical meaning is assigned to them [6]. This requirement is relevant to dementia because the clinical and imaging profiles vary between disorders [7].

Innovative computational models designed for dementia detection focus on combining efficiency with clarity [8]. Such models aim to uncover nuanced patterns of brain activity through rigorous feature extraction and robust classification frameworks, contributing to more precise and interpretable diagnoses [9]. This emphasis on explainability enables these models to serve as practical tools in clinical settings and foster collaboration between technology and healthcare [4].

The literature includes different machine learning-based dementia detection methods using EEG signals. Below are some of the current ones.

Adebisi et al. [10] developed a framework using EEG-based brain functional networks to differentiate dementia-related disorders using EEG data from 50 subjects. They obtained an accuracy of 95.07% in the delta band with an 80:10:10 split ratio. Nour et al. [11] proposed a model for AD classification using EEG data from 104 AD patients and 36 healthy controls. They achieved 97.9% accuracy with a five-fold CV. Kim et al. [12] classified the Alzheimer’s spectrum using EEG from 58 participants. They calculated 93.10% accuracy during memory encoding with an 80:20 split ratio. Cao et al. [13] proposed a framework to detect dementia using EEG data from 60 participants. They obtained an accuracy of 93.00% using an 80:20 split ratio. Yu et al. [14] integrated EEG and genetic data for AD classification using data from 143 participants. They attained 93.00% accuracy with an 80:20 split ratio and five-fold CV.

Recent studies on the same public EEG cohort have examined complementary representations. Ghassemkhani et al. [15] analyzed spatial complexity and spectral signatures. Schwerin et al. [16] evaluated spectral and processed-monitoring indices. Jungrungrueang et al. [17] used dynamic EEG connectivity with subject-wise cross-validation. These studies support the need to distinguish physiological feature analysis from held-out diagnostic classification.

Mlinarič et al. [18] evaluated functional-connectivity classifiers with leave-one-subject-out validation. Liu and Meng [19] studied a dynamic-threshold graph convolutional network on the same dataset. Kang et al. [20] examined clustered pattern projections and their reliability under subject-wise evaluation. Validation level, endpoint, and participant aggregation must be considered before methods are compared.

### 1.1. Literature Gaps

In the previous section, a literature review was provided. According to this review, the identified gaps in the literature are as follows:

- Deep learning has been applied to EEG classification [21]. Its computational demands and validation requirements depend on the model and evaluation design [22]. Participant separation is essential in translational EEG studies because segment overlap can inflate apparent generalization [23].
- Existing models for EEG signal classification and dementia detection have generally prioritized achieving high classification performance [10], but they often lack explainability.
- There is a scarcity of studies that offer visual interpretations [24], such as connectome-style diagrams, to show which scalp regions contribute to the decisions of dementia detection models.

### 1.2. Motivation and Our Model

The main motivation behind the proposed PNPat-based research is to address the identified gaps in the literature concerning dementia detection and EEG signal classification. Specifically, the following:

The main objective is to develop an interpretable symbolic feature representation and to assess its behavior under participant-independent validation. BCT and PNPat provide the feature extraction stage. This stage is linear in signal length for a fixed channel montage. The costs of supervised selection and classification are reported separately.

Existing models in the field often lack explainability, a critical factor in clinical applications. To address this, we have integrated the Directed Lobish (DLob) [25] method with the proposed PNPat-based feature engineering model, resulting in a self-organized explainable feature engineering (SOXFE) model. This model is self-organized because it can automatically select the best outcomes from the generated results. It is an explainable feature engineering (XFE) model because it extracts interpretable results using the integrated DLob method. By calculating the complexity and transition tables of the generated DLob sentences, we aimed to obtain outputs that are interpretable at the feature level.

DLob sentences are used to display the scalp-region transitions retained by the selector. The resulting diagrams provide an inspectable account of the feature representation. They do not measure functional or effective connectivity. A clinical or neurophysiological interpretation requires independent validation.

The general structure of the proposed model is outlined as follows:

- Feature Extraction: The primary objective in this phase is to uncover hidden channel transitions using two novel methods: (i) Bipartite Channel Transformer (BCT) and (ii) Previous-Next Pattern (PNPat). By applying BCT, two transformed signals are generated by sorting in descending and ascending order. The hidden transitions are then extracted using the PNPat feature extraction method, which identifies both previous and next transitions. As a result, four feature vectors are obtained during this phase.
- Feature Selection: In this phase, we employ the cumulative weight-based iterative neighborhood component analysis (CWINCA) [26] feature selector. This method is used to refine the four feature vectors generated in the extraction phase, producing four selected feature vectors.
- Explainable Results Generation: Also referred to as the Explainable AI (XAI) phase, this stage involves generating DLob sentences for each selected feature by using the DLob symbolic language and the indices of the selected features. By analyzing these DLob sentences, explainable results are produced, providing insights into the model’s decisions.
- Classification: The tkNN ensemble evaluates a defined grid of nearest-neighbor settings. Iterative majority voting (IMV) combines candidate outputs. The final configuration is selected within the training data. Four classifier-based outcomes are generated.
- Information Fusion: Combinational majority voting (CMV) combines the four classifier-based outcomes. The candidate combinations and the final selection rule are defined below. Under participant-independent evaluation, all configuration choices are confined to training participants.

### 1.3. Innovations and Contributions

**Innovations:**

- A new channel transformer, termed BCT, has been proposed. BCT generates two transformed signals by applying both ascending and descending sorting functions.
- A new transition-based feature extraction function, termed PNPat, has been introduced.
- By combining the proposed BCT and PNPat for feature extraction, CWINCA for feature selection, tkNN for classification, DLob for interpretable results generation, and CMV for information fusion, a new SOXFE model has been developed.

**Contributions:**

- The methodological contribution is a symbolic feature engineering framework based on BCT and PNPat, with established selection, classification, and voting components. The findings support the feasibility of interpretable, algorithm-derived representations. They do not establish reproducible disease-related EEG characteristics. The evaluation also documents the substantial dependence of performance on participant separation and model-selection procedures.
- DLob maps selected transition indices to scalp-region symbols. These outputs provide feature-level traceability. The present results do not validate the symbols as biomarkers of cortical dysfunction or establish an accuracy advantage for symbolic mapping.

## 2. EEG Dementia Dataset

In this research, a publicly available EEG dementia dataset [27] has been utilized. This dataset contains three classes: (1) AD, (2) frontotemporal dementia (FD), and (3) Healthy. The data was collected from 88 participants using a 19-channel brain cap. The channels used are: (1) Fp1, (2) Fp2, (3) F7, (4) F3, (5) Fz, (6) F4, (7) F8, (8) T3, (9) C3, (10) Cz, (11) C4, (12) T4, (13) T5, (14) P3, (15) Pz, (16) P4, (17) T6, (18) O1, and (19) O2. The names of these channels are crucial for creating the DLob string. Moreover, the channels were positioned according to the 10/20 system. The cognitive state of each participant was assessed with the Mini-Mental State Examination (MMSE); the mean MMSE scores were 17.75 for AD, 22.17 for FD, and 30 for Healthy. MMSE scores describe the degree of cognitive impairment and were not used to define the diagnostic labels. The EEG signals were divided into non-overlapping 15-second segments to create the dataset. The sampling frequency of the brain cap used was 500 Hz, resulting in a segment length of 7500 samples (500 × 15). The properties of this dataset are also demonstrated in Table 1 to clarify the EEG dementia dataset used.

**Clinical characteristics.** The recordings were acquired during routine resting-state, eyes-closed EEG examinations at the 2nd Department of Neurology of AHEPA General University Hospital of Thessaloniki, Greece, where the diagnoses were established clinically by an experienced team of neurologists [27]. The public release provides the diagnostic group, sex, age, and MMSE score of each participant; biomarker confirmation, frontotemporal dementia subtype, and medication data are not provided. The median disease duration was 25 months (interquartile range, 24–28.5 months), and no dementia-related comorbidities were reported for the AD group [27]. The mean MMSE values (AD 17.75 ± 4.50; FD 22.17 ± 8.22) indicate mainly moderate-stage impairment in AD and heterogeneous impairment in FD; the cohort therefore does not represent preclinical or prodromal stages. The recordings were made with a Nihon Kohden EEG 2100 device with 19 scalp electrodes placed according to the 10–20 system and two mastoid electrodes (A1, A2) at a sampling frequency of 500 Hz. The total recording time was 485.5 min for AD, 276.5 min for FD, and 402.0 min for healthy participants. The dataset also provides preprocessed recordings, obtained by 0.5–45 Hz Butterworth band-pass filtering, re-referencing to A1–A2, artifact subspace reconstruction, and independent component analysis with automatic rejection of eye and jaw components [27]. In this study, the preprocessed recordings were divided into non-overlapping 15 s segments (7500 samples). Because the number of segments per participant depends on recording length, the class distribution differs at the segment level (AD 41.7%, FD 23.7%, Healthy 34.6%) and at the participant level (AD 40.9%, FD 26.1%, Healthy 33.0%).

## 3. The Proposed SOXFE Model

The primary objectives of the proposed SOXFE model are:

- Evaluating classification performance under participant-independent protocols, with explicit comparison against a majority-class baseline.
- Presenting explainable results.

To fulfill these objectives, we have developed a new SOXFE model with five main phases: (i) multiple feature extraction using the proposed BCT and PNPat, (ii) selection of the most meaningful/informative features using CWINCA, (iii) generation of DLob-based XAI results, (iv) classifier-based outcome generation—classification—using the tkNN classifier, and (v) information fusion based on CMV and a greedy technique.

In the first phase, EEG signals are converted into channel identities using BCT, resulting in two transformed signals. The proposed PNPat serves as the essential feature extractor of the SOXFE model. Two feature vectors are extracted from each transformed signal using the PNPat feature extractor, yielding four feature vectors.

In the feature selection phase, an iterative feature selector is required. While INCA is a well-known method, determining the range of INCA can be challenging. Therefore, CWINCA has been utilized in this research to select the most informative features, resulting in four selected feature vectors. The outputs of the feature selection phase are used in both the generation of XAI results and the classification phase.

To achieve high classification performance, we have employed an ensemble classifier, tkNN. By applying the tkNN classifier to the selected features, four classifier-based outcomes have been generated.

In the final phase, information fusion, CMV is applied to the four classifier-based outcomes, creating four additional voted outcomes. The best of these eight outcomes is then selected using a greedy technique.

**Figure 1:**
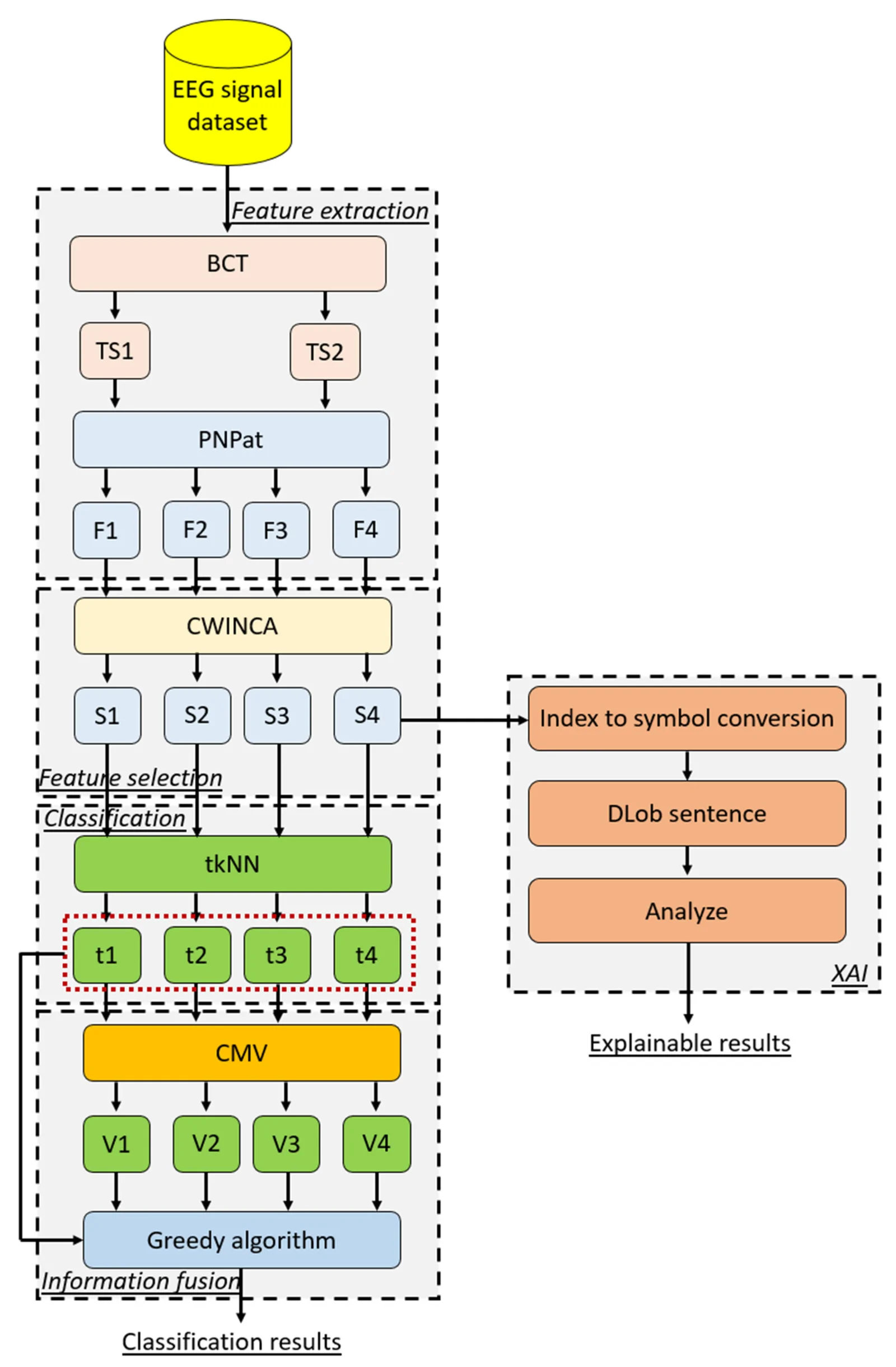
Graphical overview of the proposed BCT and PNPat-based SOXFE model. Herein, TS: transformed signal, F: feature vector, S: selected feature vector, t: classifier-based outcome, V: voted outcome.

The graphical overview of the proposed BCT and PNPat-based SOXFE model is illustrated in Figure 1.

The details of the presented SOXFE model are given below.

### 3.1. Feature Extraction

The first phase of the proposed SOXFE model is feature extraction. In this phase, the primary objective is to detect hidden and informative channel transitions. To achieve this, two new methods have been introduced: (i) BCT and (ii) PNPat. By utilizing BCT, two transformed signals have been generated, each containing information about the channels. The features of these transformed signals are then extracted using PNPat, resulting in four feature vectors. Each feature vector has a length of 361 (=19 × 19) since the EEG dataset used has 19 channels. The steps of this phase are as follows:

Step 1 (BCT): Apply the recommended BCT to EEG signals and create transformed signals.

(1) $${chn}^{i}=signal\left(1:19,i\right),\;i\in \{1,2,\ldots ,len\}$$

(2) $$id_{i}^{1}=argsort({chn}^{i})$$

(3) $$id_{i}^{2}=argsort(-{chn}^{i})$$

(4) $$ts_{q}\left(c:c+18\right)=id_{i}^{q},\;c\in \left\{1,20,\ldots ,19len-18\right\},\;q\in \{1,2\}$$

Above, the mathematical definition of the proposed BCT has been defined, and two transformed signals have been used, where ${chn}^{i}$: channels vector of the ith EEG signal (signal), len: length of the EEG signal, argsort(.): sorting function, $id^{1}$: indices of the sorted channels in ascending order, $id^{2}$: indices of the sorted channels in descending order and ts: transformed signal with a length of 19len since the used EEG signal dataset has 19 channels.

Step 2 (PNPat): Extract features from transformed signals employing the proposed PNPat feature extractor. The mathematical expression of the presented PNPat is given below.

(5) $$tt_{1}=tt_{2}=tt_{3}=tt_{4}=\left[\begin{array}{ccc} 0 & \ldots & 0\\ \vdots & \ddots & \vdots \\ 0 & \ldots & 0 \end{array}\right]$$

(6) $$tt_{1}\left(ts_{1}\left(j\right),ts_{1}\left(j-1\right)\right)=tt_{1}\left(ts_{1}\left(j\right),ts_{1}\left(j-1\right)\right)+1,\;j\in \{2,3,\ldots ,19len\}$$

(7) $$\begin{array}{c} tt_{2}\left(ts_{1}\left(k\right),ts_{1}\left(k+1\right)\right)=tt_{2}\left(ts_{1}\left(k\right),ts_{1}\left(k+1\right)\right)+1,\;k\\ \in \{1,2,\ldots ,19len-1\} \end{array}$$

(8) $$tt_{3}\left(ts_{2}\left(j\right),ts_{2}\left(j-1\right)\right)=tt_{3}\left(ts_{2}\left(j\right),ts_{2}\left(j-1\right)\right)+1,$$

(9) $$tt_{4}\left(ts_{2}\left(k\right),ts_{2}\left(k+1\right)\right)=tt_{4}\left(ts_{2}\left(k\right),ts_{2}\left(k+1\right)\right)+1,$$

where tt: transition table with a size of 19 × 19. Above, we have created the transition tables of the transformed signals. Then, we applied the summation-based normalization to these four transition tables.

(10) $$tt_{g}^{N}\left(a,b\right)=\frac{tt_{g}\left(a,b\right)}{\sum_{b=1}^{19}tt_{g}\left(a,b\right)},\;g\in \left\{1,2,\ldots ,4\right\},\;a,b\in \left\{1,2,\ldots ,19\right\}$$

Herein, $tt^{N}$: the normalized transition table. In the third step, the normalized transition tables have been converted to a vector to obtain a feature vector.

(11) $$f_{g}\left(z\right)=tt_{g}^{N}\left(a,b\right),\;z\in \left\{1,2,\ldots ,361\right\}\;$$

Here, f: feature vector with a length of 361 (19 × 19), and Equation (11) defines the matrix-to-vector transformation.

Step 3 (Feature matrix generation): Implement Steps 1–2 until the number of EEG signals is reached, and generate four feature matrices.

### 3.2. Feature Selection

CWINCA [26] applies iterative feature selection within a range determined from cumulative NCA weights. The normalized feature matrix is ranked by supervised NCA. Candidate subset sizes are evaluated within the selected range. The definitions below specify the procedure without requiring a separate citation for the iterative loop.

Step 4 (Feature selection with CWINCA): Choose the most informative/meaningful features of the feature matrix generated by deploying CWINCA.

(12) $$sf_{g}=\psi (X_{g},y)\;$$

where sf: selected feature vector, ψ(.): CWINCA feature selector, X: feature matrix generated and y: actual label.

The CWINCA function serves as the main feature selector. Two constant values have been used: 0.85 (for detecting the start value) and 0.9999 (for detecting the stop value) to define the range of the loop. The steps of the CWINCA process are also outlined below.

Step 4.1: Compute the qualified indexes of the feature matrices by using the NCA [28] feature selector.

(13) $$X_{g}^{N}\left(:,x\right)=\frac{X_{g}\left(:,z\right)-\min\left(X_{g}\left(:,z\right)\right)}{\max\left(X_{g}\left(:,z\right)\right)-\min\left(X_{g}\left(:,z\right)\right)+\varepsilon }\;,\;z\in \left\{1,2,\ldots ,361\right\}$$

(14) $$[wg_{g},{qin}_{g}]=NCA(X_{g},y)\;$$

Herein,$\;X^{N}$: normalized feature matrix deploying min-max normalization, ε: a tiny value, qin: qualified indexes of the features and wg: weights of the features.

Step 4.2: Calculate start and stop values utilizing cumulative weights.

(15) $$cw^{g}=\frac{\sum_{c=1}^{i}wg_{g}\left({qin}_{g}\left(c\right)\right)}{\sum_{c=1}^{361}wg_{g}\left({qin}_{g}\left(c\right)\right)},\;i\in \{1,2,\ldots ,361\}$$

(16) $$start_{g}=i,\;cw^{g}(i)>0.85,\;$$

(17) $${stop}_{g}=i,\;cw^{g}\left(i\right)\leq 0.9999,$$

where cw: cumulative weight.

Step 4.3: Apply an iterative feature selection process using the computed start and stop values and compute the loss value of each selected feature vector.

(18) $$sf_{g}^{q}\left(d,h\right)=X_{g}^{N}\left(d,qin_{g}(h)\right),\;h\in \left\{1,2,\ldots ,q\right\},$$

(19) $$\;d\in \left\{1,2,\ldots ,ns\right\},\;q\in \left\{start_{g},start_{g}+1,\ldots ,{stop}_{g}\right\}$$

(20) $$loss_{g}\left(q-start_{g}+1\right)=kNN(sf_{g}^{q},y)$$

Herein, sf: selected feature vector, ns: number of signal and loss: misclassification rate of the selected feature vector.

Step 4.4: Choose the best selected features among the candidate selected feature vectors by using the minimum misclassification rate.

(21) $$ix_{g}=argmin(loss_{g})$$

(22) $$S_{g}=sf_{g}^{ix_{g}+start_{g}-1}$$

where ix: the indices of the minimum loss value and S: final/ultimate selected feature. Moreover, to extract the DLob symbol, we need to memorize indices of the selected features as below.

(23) $$sind_{g}\left(w\right)={qin}_{g}\left(w\right),\;w\in \left\{1,2,\ldots ,ix_{g}+start_{g}-1\right\}$$

Herein, sind: selected feature indices.

### 3.3. Explainable Results Generation

To extract interpretable results, the presented model uses DLob, a symbolic language in which each symbol represents specific information about the lobes. The DLob symbols have been applied according to the brain cap used in this study, and the meanings of these DLob symbols for the brain cap are detailed in Table 2.

DLob strings are generated from the symbols in Table 2 and the selected transition indices. BCT and PNPat encode each feature as a pair of channel identities. DLob maps these identities to scalp-region symbols. The resulting sentences, symbol entropies, and transition diagrams describe the selected representation. They are not direct measurements of brain condition.

Step 5 (DLob-based explainable results generation): Generate the DLob sentences by deploying selected feature indices.

(24) DL={FL,FR,FL,FL,Fz,FR,FR,TL,CL,Cz,CR,TR,TL,PL,Pz,PR,TR,OL,OR}

(25) $$val_{1}=\left\lceil \frac{sind_{g}\left(t\right)}{19}\right\rceil ,\;t\in \{1,2,\ldots ,N\}$$

(26) $$val_{2}=(sind_{g}\left(t\right)-1)\;\left(mod\;19\right)+1$$

(27) $$snt_{g}\left(c\right)=DL\left(val_{1}\right),\;c\in \{1,3,\ldots ,2N-1\}$$

(28) $$snt_{g}\left(c+1\right)=DL\left(val_{2}\right)$$

Herein DL: Look up table of the DLob, val: the indices of the used LUT and snt: DLob sentence. The look-up table contains one symbol for each of the 19 channels in the channel order given in the dataset section (Fp1, Fp2, F7, F3, Fz, F4, F8, T3, C3, Cz, C4, T4, T5, P3, Pz, P4, T6, O1, O2). For a selected feature index z ∈ {1, 2, …, 361}, Equations (25) and (26) return the two channel indices of the transition encoded by z, both in {1, 2, …, 19}; Equation (26) has been corrected to ((z − 1) mod 19) + 1 so that the 361 indices map one-to-one onto the entries of the 19 × 19 transition table in Equation (11).

Using the extracted DLob sentences, we calculated the entropy values and created histograms of the symbols used. Additionally, connectome-style transition diagrams were generated to visualize the channel transitions of the selected features. Through this phase, explainable results were produced.

### 3.4. Classification

To generate classifier-based results, we utilized an ensemble classifier known as tkNN. The tkNN classifier generates multiple outcomes by iteratively changing parameters, and IMV is employed to produce voted outcomes. In the final step, the most accurate outcome is selected using a greedy algorithm. In this phase, four classifier-based outcomes were generated, as tkNN uses the four selected features as inputs. The classification step is depicted below.

Step 6 (Classification with tkNN): Classify the selected features by deploying tkNN classifier and generate classifier-based outcomes.

(29) $${co}_{g}=\phi (S_{g},y)\;$$

where co: selected classifier-based outcome and ϕ(.): tkNN classifier.

The steps of the used tkNN classifier are also depicted below.

Step 6.1: Generate parameter-based outcomes by using the iterative parameter changing method.

(30) Dist={cityblock,euclidean,cosine}

(31) wg={squaredinverse,equal} 

(32) kv={1,2,…,5} 

(33) $$po_{e}^{g}=kNN\left(S_{g},y,Dist_{a},wg_{b},kv_{c}\right),\;$$

(34) $$a\in \left\{1,2,3\right\},\;b\in \left\{1,2\right\},\;c\in \left\{1,2,\ldots ,5\right\},\;e\in \left\{1,2,\ldots ,30\right\}$$

where Dist: distance metric, wg: weight and kv: k value parameters. By changing these parameters iteratively, 30 parameter-based outcomes (po) are generated for each selected feature vector.

Step 6.2: Compute voted outcomes deploying the iterative majority voting (IMV) method to parameter-based outcomes.

(35) $$acc(e)=\theta \left(po_{e}^{g},y\right)$$

(36) index=argsort(−acc) 

(37) $${vo}_{l}^{g}=\varpi \left(po_{index\left(1\right)}^{g},\;po_{index\left(2\right)}^{g},\ldots ,po_{index\left(l+2\right)}^{g}\right),\;l\in \left\{1,2,\ldots ,28\right\}\;$$

Here, acc: classification accuracy, θ(.): classification accuracy computation function, index: the qualified indexes of the classification accuracy in descending order, ϖ(.): mode function and vo: voted outcome. Herein, the iteration range of IMV is from 3 to 30. Therefore, 28 voted outcomes have been created in this step.

Step 6.3: Select the ultimate outcome among the 58 (=30 parameter-based + 28 voted) outcomes deploying the greedy algorithm.

(38) $$acc(30+l)=\theta \left({vo}_{l}^{g},y\right)$$

(39) id=argmax(acc) 

(40) $$cout^{g}=\left\{\begin{array}{c} po_{id}^{g},\;id\leq 30\\ vo_{id-30}^{g},\;id>30 \end{array}\right.$$

where id: index of the maximum classification accuracy and $cout^{g}$: gth final outcome.

### 3.5. Information Fusion

The final phase of the presented model is information fusion. In this phase, we applied the CMV algorithm to the four generated outcomes, resulting in four voted outcomes. The primary objective of this phase is to enhance the classification ability of the proposed model. The step involved in the information fusion phase is outlined below:

Step 7 (Information fusion): Generate voted outcomes using the CMV algorithm and select the most accurate outcome as the final result.

(41) $$vo^{1}=\varpi \left(cout^{1},cout^{2},cout^{3}\right)$$

(42) $$vo^{2}=\varpi \left(cout^{1},cout^{3},cout^{4}\right)$$

(43) $$vo^{3}=\varpi \left(cout^{2},cout^{3},cout^{4}\right)$$

(44) $$vo^{4}=\varpi \left(cout^{1},cout^{2},cout^{3},cout^{4}\right)$$

As can be seen above, by applying the combinational mode function (CMV), four voted results were generated using the four classifier-based outcomes, and the best result (the outcome with the highest classification accuracy) was selected using the greedy algorithm.

(45) $$acc\left(r\right)=\theta \left(cout^{r},y\right),\;r\in \left\{1,2,\ldots ,4\right\}\;$$

(46) $$acc\left(r+4\right)=\theta \left(vo^{r},y\right)$$

(47) id=argmax(acc) 

(48) $$fout=\left\{\begin{array}{c} cout^{id},\;id\leq 4\\ {vo}^{id-4},\;id>4 \end{array}\right.$$

where fout: final outcome of the proposed model.

The seven steps given above define the proposed BCT and PNPat-based SOXFE model.

## 4. Performance Evaluation

To obtain the results of the proposed model, we implemented the recommended model on a simple configured laptop using MATLAB (version 2024a). The presented BCT and PNPat-based model was implemented by creating functions, all of which were stored as .m files. The asymptotic cost of each phase is given below, followed by the measured run times.

The first phase is feature extraction, and the complexity of this phase is computed below using Big O notation.

(49) $$t_{BCT}=O(2CL)≅O(CL)\;$$

(50) $$t_{PNPat}=O(4CL)≅O(CL)$$

(51) $$t_{FE}=O\left(CL+CL\right)≅O(CL)$$

Herein, $t_{BCT}$: time complexity of the BCT, C: number of channels, L: length of the EEG signal, $t_{PNPat}$: time complexity of the PNPat and $t_{FE}$: time complexity of the recommended feature extraction function. In this case, the time complexity of the feature extraction is equal to O(CL).

Sorting the C channel values at each time point costs O(C log C). Because C = 19 is fixed by the montage, this factor is treated as a constant in Equation (49); the feature extraction cost is therefore linear in the signal length L, and it is independent of the labels.

In the feature selection phase, the CWINCA feature selector has been used. Therefore, we have computed the time complexity of the CWINCA. In order to compute the time complexity of the CWINCA, the time complexities of NCA and kNN have been used, and the formulation is given below.

(52) $$t_{NCA}=O(N)\;$$

(53) $$t_{LC}=O(RK)$$

(54) $$t_{CWINCA}=O(N+RK)$$

Herein, N: time complexity coefficient of the NCA, R: range of the feature selection loop, K: time complexity of the kNN classifier and LC: loss value computation. The time complexity of the CWINCA ($t_{CWINCA}$) is the addition of the time complexity of the NCA ($t_{NCA}$) and loss computation ($t_{LC}$). Therefore, the time complexity of this phase is O(N+RK).

The terms N and K hide the dependence on the number of training observations n and the number of features d. NCA is optimized iteratively, and each iteration requires the pairwise distances between observations, i.e., O(n^2^d) operations. Each kNN evaluation also requires O(n^2^d) distance computations, and CWINCA repeats this evaluation R times. Hence, N and K grow quadratically with n.

The third phase of the presented model is the XAI. In this phase, the indices of the selected features are used in a loop to generate the DLob sentences. The time complexity of this phase is

(55) $$t_{DLob}=O(P)\;$$

where P: the length of selected feature indices.

tkNN is used to compute classification outcomes. The essential functions of the tkNN are parameter-based outcome generations and IMV. In this aspect, the time cost of this function is

(56) $$t_{POG}=O(RK)\;$$

(57) $$t_{IMV}=O(R)$$

(58) $$t_{tkNN}=O(R+RK)$$

Herein, $t_{POG}$: time complexity of the parameter-based outcome generation and $t_{IMV}$: cost of the IMV algorithm.

The last phase is the information fusion phase, which generates eight outcomes (=4 classifier-based + 4 voted). Therefore, the time complexity of this phase is

(59) $$t_{IF}=O(H)$$

Here, $t_{IF}$: cost of the information fusion and H: the length of the outcomes.

In this aspect, the total time cost of the proposed SOXFE model is given below.

(60) $$t_{Total}=O\left(CL+N+RK+R+RK+H\right)≅O(CL+N+RK+H)$$

where $t_{Total}$ is the total time complexity of the recommended SOXFE model, and it is O(CL+N+RK+H). Equation (60) is linear only in the signal length L. Because N and K grow quadratically with the number of observations, and the R CWINCA iterations and the 30 tkNN parameter settings multiply the kNN cost, the complete training procedure is not linear in sample size; for n observations and d selected features, its cost is dominated by O((R + 30)·n^2^·d).

**Measured run times.** The run times were recorded for Case 1 (4661 segments) with MATLAB 2024a on a laptop with 32 GB RAM. Feature extraction required 0.012 s per segment (56 s for all segments and the four feature vectors). CWINCA required 1500 s for the four feature vectors, and tkNN with IMV required 60 s. The total training time was 1650 s, of which CWINCA accounted for approximately 91%. Classification of a new segment with the trained model required 0.020 s.

The recommended SOXFE model is an EEG classification model. Therefore, we used the following evaluation metrics for assessing classification results: classification accuracy, geometric mean (GM), balanced accuracy (BA), sensitivity (recall), specificity, precision (positive predictive value), negative predictive value (NPV), F1-score, and macro-averaged F1-score. Confusion matrices are reported for each case, and 95% confidence intervals (CIs) for accuracy were calculated with the Wilson score method. For the binary cases, the positive classes were dementia (Case 2), AD (Cases 3 and 4), and FD (Case 5); for Case 1, class-wise metrics were calculated in a one-versus-rest manner. Additionally, to demonstrate the general classification performance of the proposed model and to extract DLob transition diagrams for each case, five cases were defined. These cases are as follows:

Case 1: Classification of AD vs. FD vs. Healthy. This case contains three classes.

Case 2: Classification of dementia or not. This case has two classes, and these are (AD + FD) vs. healthy.

Case 3: Classification of AD vs. FD. The objective of this case is to extract differences between these two disorders.

Case 4: Classification of AD vs. Healthy. This case is termed AD detection.

Case 5: Classification of FD vs. Healthy. This case is termed FD detection.

The original evaluation used segment-wise 10-fold cross-validation without participant grouping. Segments from the same participant were therefore not constrained to a single fold and could occur in both training and test sets. These results describe segment classification under a participant-mixed protocol.

Generalization to unseen participants was assessed separately with leave-one-subject-out (LOSO) cross-validation. In each iteration, all segments from one participant were held out, and the remaining participants formed the training set. Segment-level LOSO accuracy was calculated from the pooled predictions for the held-out segments. Participant-level accuracy was calculated separately after a majority vote across each held-out participant’s segment predictions.

A participant-grouped outer cross-validation procedure was used to assess generalization. All segments from an outer-test participant remained outside every fitting and selection step. In the nested implementation, min–max normalization and the NCA/CWINCA ranking were fitted once on each outer training set. They were not refitted in its inner splits. Inner participant-grouped validation selected the feature count, tkNN settings, IMV/CMV configuration, and final output. This design protects the outer test set, but its inner selection scores can be optimistic because the supervised ranking is shared across inner splits [29].

The segment-wise analysis in Table 3 used the full segment set for normalization, feature ranking, and configuration selection. Its results remain a participant-mixed secondary analysis. Algorithm A1 specifies the participant-grouped implementation. Its nested segment-level metrics are calculated from pooled predictions across all outer test folds. Participant-level metrics are calculated separately after a majority vote over each held-out participant’s segment predictions. Outer-test labels were used only for final scoring.

The majority-class baseline assigns every observation to the most frequent class at the corresponding reporting level. Accuracy and balanced accuracy are reported separately for segments and participants. SOXFE and baseline correctness were compared on the same held-out participants with a two-sided exact McNemar test [30]. Here, b counts participants classified correctly only by SOXFE, and c counts those classified correctly only by the baseline. Paired differences in accuracy and balanced accuracy were summarized with participant bootstrap confidence intervals using 10,000 resamples and random seed 1. The same sampled participants were used for both methods in each resample.

Wilson confidence intervals [31] use the observation count at the stated reporting level. Segment-based intervals do not account for within-participant dependence and are interpreted descriptively. Participant-level intervals and paired participant-bootstrap comparisons provide the relevant uncertainty summaries for participant-level decisions. The nested confusion matrices were counted from held-out outer-fold classifications.

The recommended BCT and PNPat-based model generates both classifier-wise and voted outcomes for each case. Therefore, classifier-based and voted results have been depicted in Table 3.

Table 3 presents the original segment-wise results without participant grouping. The best accuracies for Cases 1–5 were 99.12%, 99.23%, 99.74%, 99.30%, and 99.56%, respectively. These values do not establish performance on unseen participants. Table 4 compares these results with the participant-independent evaluations for all five classification tasks.

Table 4 shows the substantial decrease under participant separation. Nested pooled segment accuracies were 49.00%, 62.50%, 55.99%, 61.51%, and 56.51% for Cases 1–5. Their balanced accuracies were 45.96%, 57.64%, 52.15%, 61.27%, and 54.24%. After participant-majority voting, nested accuracies were 51.14%, 65.91%, 55.93%, 64.62%, and 55.77%. Participant-majority voting did not remove the limited participant-independent discrimination.

Table 5 reports the paired participant-level comparison with the majority-class baseline. Accuracy differences and balanced-accuracy differences are expressed in percentage points.

Table 5 shows no McNemar p value below 0.05. In Case 2, LOSO participant accuracy equaled the majority-class baseline at 67.05%; the paired accuracy difference was 0.00 percentage points. In Case 3, LOSO participant accuracy was below the baseline, with a difference of −3.39 percentage points. The LOSO balanced-accuracy difference intervals excluded zero only for Cases 1, 2, and 4. Under nested validation, this occurred only for Cases 1 and 4. These balanced-accuracy findings provide limited evidence beyond the class distribution in those tasks, but they do not establish an accuracy improvement or clinical utility.

Table 6 reports the class-wise metrics under each validation protocol, and Table 7 gives the corresponding confusion matrices.

Under LOSO, the sensitivity for FD in Case 1 was 27.99%, and 477 of the 1104 FD segments (43.2%) were assigned to AD. In Case 2, the sensitivity for dementia was 74.99%, whereas the specificity was 42.69%, indicating a bias toward the larger dementia class. In Case 3, the specificity for FD was 39.31%. At the participant level, five of the 23 FD participants were correctly identified in Case 1, and 12 of the 29 healthy participants were correctly identified in Case 2 (Table 7). These results do not support diagnostic use of the model.

Per-participant LOSO accuracy, defined as the proportion of correctly classified segments of each held-out participant, had mean values of 50.30% (Case 1; standard deviation 31.0%), 61.00% (Case 2), 55.50% (Case 3), 62.00% (Case 4), and 56.00% (Case 5). In Case 1, 47.0% of the participants had a per-participant accuracy below 50%.

CWINCA retained 168–329 of the 361 features per feature vector (mean 237.35), which corresponds to a mean dimensionality reduction of 34.25% (Table 8). The largest reduction was observed in Case 4 (40.10%) and the smallest in Case 3 (21.95%). These counts refer to the segment-wise analysis and equal half the number of symbols in the corresponding DLob sentences in Table A1 because each selected feature contributes two symbols.

The presented SOXFE model generates DLob sentences, which are displayed in Table A1. Moreover, we calculated the information entropies of these DLob sentences, and these values are provided in Table 9.

Across all cases, the entropy values fall within a relatively narrow range (2.7820–2.8964 bits), well below the maximum possible value. The maximum possible entropy, given by log_2_(13), is approximately 3.7.

These entropy values quantify the diversity of the lobe symbols in the selected feature indices. They depend on the CWINCA selection and on the number of selected features and are therefore properties of the feature representation. They should not be interpreted as measures of cortical or physiological complexity. The differences between cases were small (case means 2.7962–2.8924 bits), and no statistical test or physiological complexity measure was used to support a relation with neural dysfunction.

Consequently, the entropy analysis is reported as a descriptive summary of the DLob sentences. Establishing a link with dementia-related cortical changes would require comparison with established signal-complexity measures computed from the EEG recordings and participant-independent statistical testing.

## 5. Discussion

**Figure 2:**
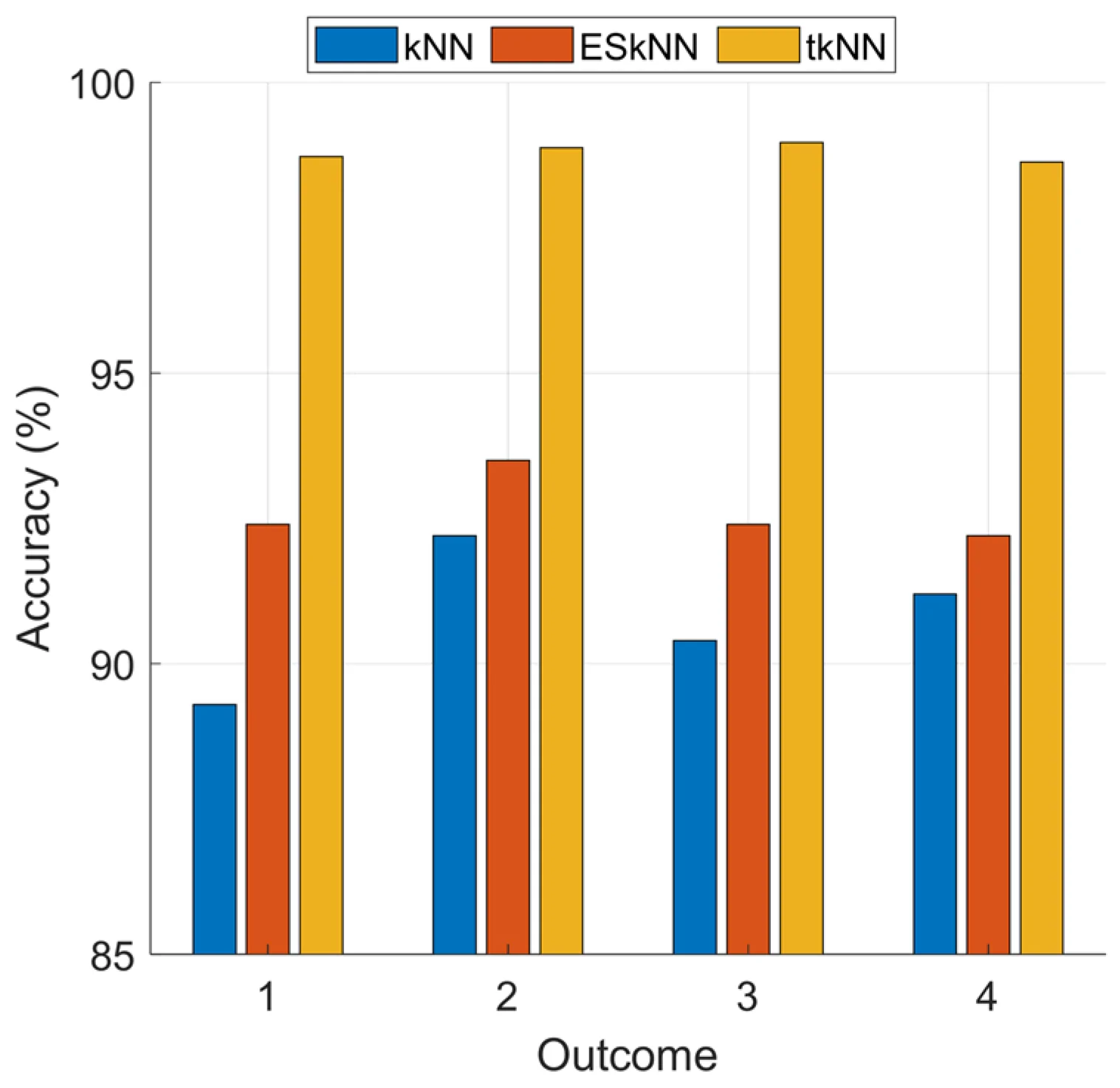
Comparative results of the kNN algorithm using outcomes of Case 1.

**Figure 3:**
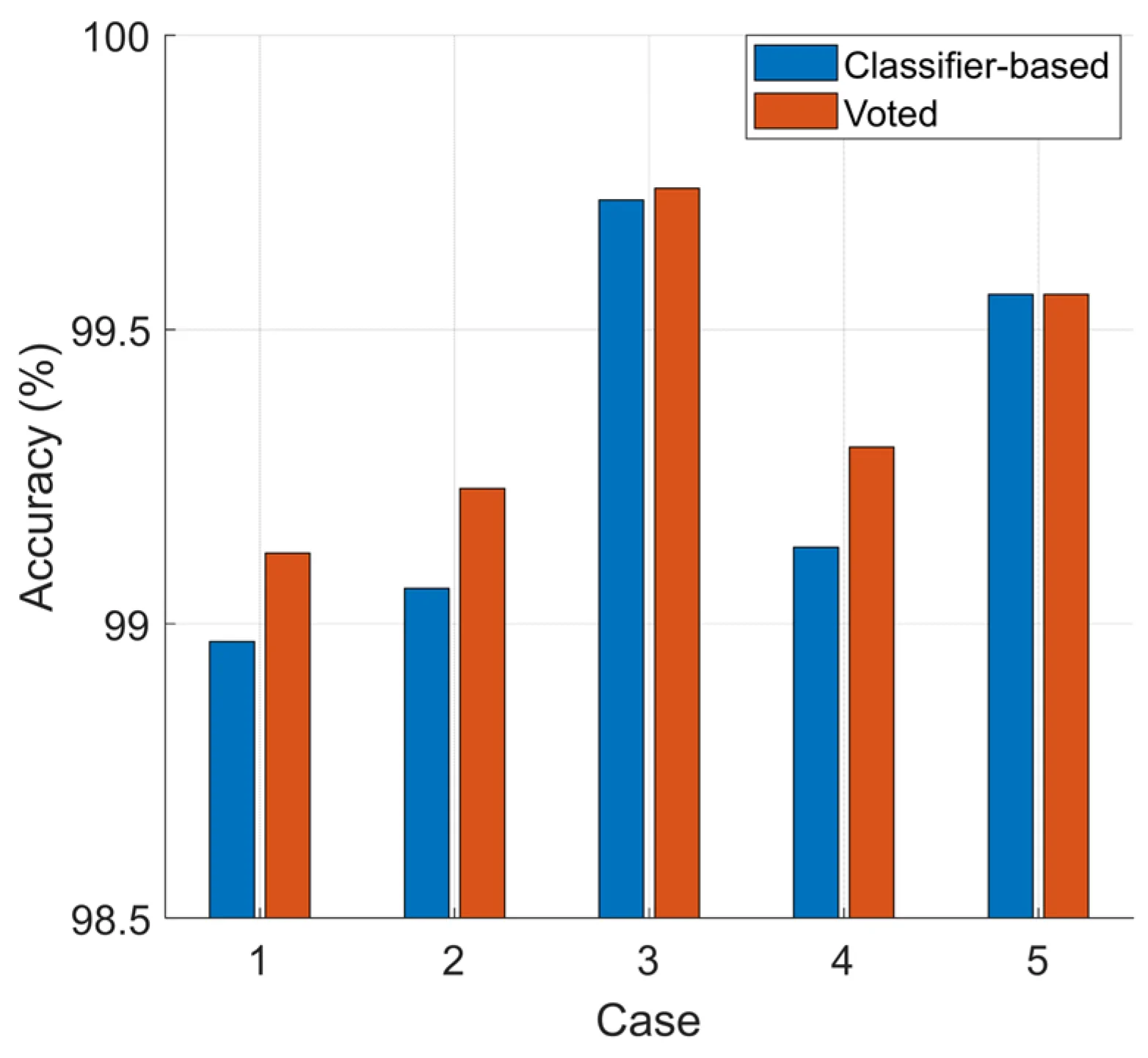
The comparative results of the best classifier-based and voted accuracies per the cases defined.

The SOXFE framework produced interpretable, algorithm-derived feature representations. The present findings do not establish reproducible disease-related EEG characteristics. Participant-independent discrimination remained limited after full reporting of the nested results and comparison with a majority-class baseline. The classifier and fusion comparisons in Figure 2 and Figure 3 concern the participant-mixed segment-wise protocol. They cannot establish diagnostic performance in unseen participants.

Under the segment-wise protocol, one factor in the classification performance of the presented model was the tkNN classifier, which is an ensemble classifier. To illustrate the effectiveness of the recommended tkNN classifier, we have depicted the classification results of kNN, ensemble subspace kNN (ESkNN), and tkNN for Case 1′s outcomes, as shown in Figure 2.

Figure 2 clearly demonstrates that tkNN achieved the best classification accuracies for all outcomes. Consequently, tkNN was utilized as the classifier in the recommended SOXFE model.

To generate the final results, we employed the CMV algorithm, with the primary objective of enhancing classification accuracy. According to the results, the use of CMV improved the classification accuracy in four out of the five cases. The comparative results of the CMV-based outcomes and classifier-based outcomes are showcased in Figure 3.

Figure 3 demonstrates that the use of CMV increased the segment-wise classification accuracies in these cases.

Studies that used the same EEG dementia dataset are summarized in Table 10, together with their validation strategies.

Table 10 shows that the reported accuracies depend strongly on the validation strategy. Studies with participant-independent validation reported 73.85–95.65%, whereas studies with random splits or k-fold cross-validation reported up to 99.64%; for the latter, participant independence was usually not specified. Under the same segment-wise protocol, the accuracy of our model (99.12%) was similar to that of N-BodyPat [32] in our re-run (97.80%). Under LOSO, both models reached only 51.19% and 52.00%, respectively, which is below the LOSO results reported in [34] (84.85%) and [35] (83.28%, binary task). Therefore, no superiority over previous methods is claimed. Sensitivity, specificity, and F1-score are not reported consistently in these studies; for our model, they are given in Table 6 and Table 7.

**Generalization across participants.** Three observations explain the gap between the segment-wise and participant-independent results. First, segment-wise accuracy was near-perfect while participant-independent accuracy was close to chance, which is consistent with PNPat transition features carrying strong participant-specific signatures; when segments of the same participant are present in the training folds, the kNN-based classifiers can match a test segment to segments of the same participant rather than to a disease-related pattern. Second, per-participant accuracies were widely dispersed (Case 1: mean 50.30%, standard deviation 31.0%; 47.0% of participants below 50%), so the errors were concentrated in a subset of participants rather than spread uniformly. Third, per-participant accuracy was not associated with disease severity (Spearman ρ = −0.12 between per-participant accuracy and MMSE in the AD and FD patients); disease severity therefore did not explain the errors. The re-run baseline [32] showed the same pattern (97.80% with segment-wise 10-fold CV and 52.00% with LOSO in Case 1), which indicates that the decrease is related to the validation protocol and the size of the dataset rather than to the SOXFE model alone. With 88 participants, 361 features per feature vector, and several supervised selection steps, the number of independent observations is small relative to the selection capacity of the model. Accordingly, the present model is a proof-of-concept for explainable feature engineering and not a clinically deployable diagnostic tool.

The majority-class comparisons qualify the apparent diagnostic value. For dementia versus healthy controls, LOSO participant accuracy equaled the majority-class rate. For AD versus FD, it fell below that rate. No exact McNemar comparison established an accuracy advantage. Balanced accuracy provided evidence beyond the class distribution only in the tasks whose paired confidence intervals excluded zero. This evidence was limited and depended on the validation protocol.

Table 11 examines the stability of the selected representation and its comparison with non-symbolic features under participant-grouped validation. The symbolic map itself does not alter the transition values supplied to the classifier. Its benefit is therefore assessed as interpretability and feature traceability, not as an assumed increase in classification accuracy.

Table 11 shows mean Jaccard values of 0.61–0.67 and transition-table correlations of 0.70–0.76. In Case 3, mean Jaccard was 0.61, below the random-subset reference of 0.62. Selection stability therefore did not consistently exceed the random reference. The non-symbolic comparator produced segment-level accuracies of 50.50%, 63.00%, 57.00%, 64.50%, and 58.00%. These exceeded the corresponding symbolic-feature accuracies in Table 4. No paired superiority test for this comparator was reported. The DLob contribution is consequently restricted to interpretation of selected indices, and no accuracy advantage or reproducible disease-specific symbolic signature is claimed.

**Figure 4:**
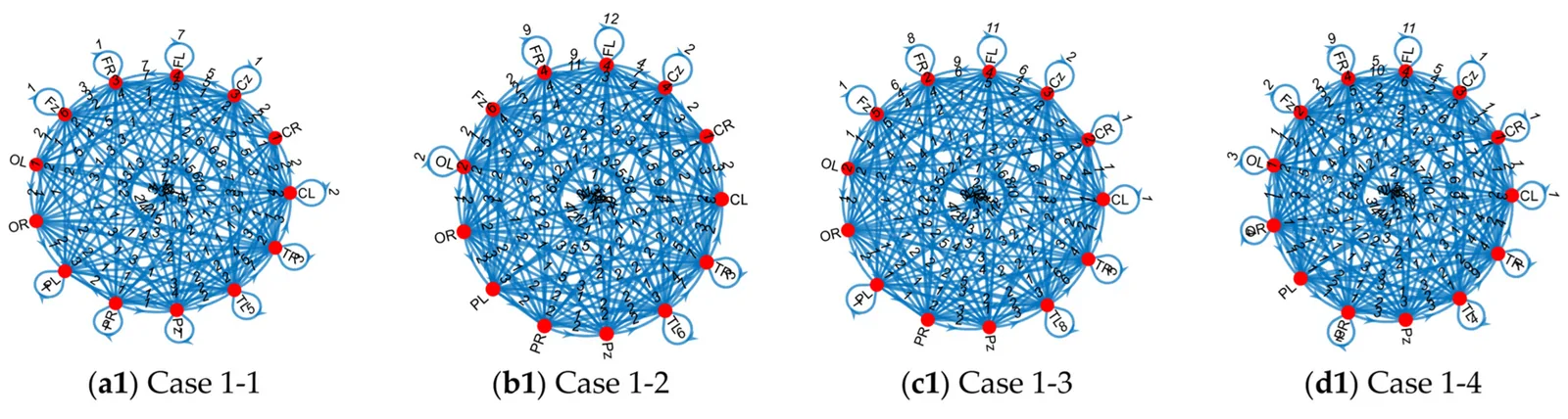

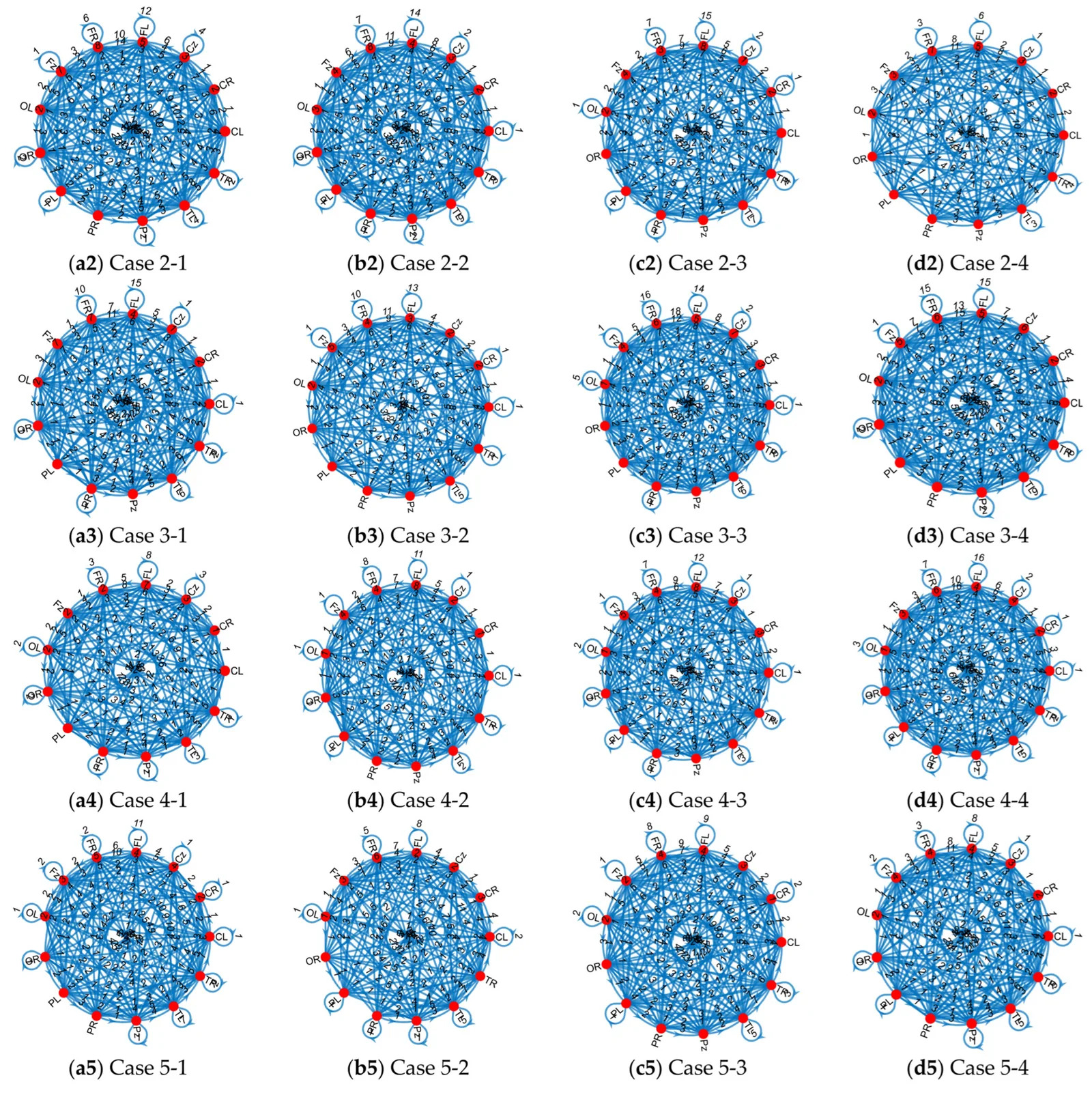
DLob feature-transition (connectome-style) diagrams generated from the outcomes of the cases. Edges denote transitions between the lobe symbols of the selected features, not functional or effective connectivity.

The SOXFE model generates explainable results with the DLob symbolic language, and connectome-style DLob transition diagrams were extracted for the outcomes of each case (Figure 4).

**Interpretation of the DLob transition diagrams.** Each edge in Figure 4 and each entry in Table 12, Table 13, Table 14, Table 15 and Table 16 counts how often a pair of lobe symbols occurs in the indices of the selected features. These transitions arise from the rank order of channel amplitudes (BCT) and from supervised feature selection. They are not estimates of functional connectivity, which quantifies statistical dependence between signals (e.g., coherence, the phase lag index [47], or the weighted phase lag index (wPLI) [48]), or of effective (directed) connectivity. To examine this distinction, the lobe-level DLob transition matrix of Case 1 was compared with lobe-level connectivity matrices computed from coherence and wPLI; the Spearman correlation was weak (ρ = 0.15). The diagrams are therefore presented as feature-driven visualizations that show which channel pairs the model relied on. Statements on regional involvement in the following paragraphs refer to these feature transitions and are hypotheses that require confirmation with established connectivity measures and participant-independent statistics. Moreover, the four transition tables of each case were averaged, and the resulting average transition tables are given in Table 12, Table 13, Table 14, Table 15 and Table 16.

Table 12 (AD versus FD versus healthy) shows the most frequent transitions between the DLob symbols of the selected features. The FL region depicts the highest transition frequencies, particularly to other frontal regions such as FR, TL, and TR, with a maximum transition value of 10.25 to FL itself, indicating that frontal–frontal channel transitions were retained most often for distinguishing between the three groups. Frontal channels therefore contributed most to the selected features. This may be compatible with the executive dysfunction reported in AD and FD, and the left frontal predominance with speech-motor involvement in some FD variants, but the transitions do not demonstrate frontal dysfunction, and FD subtypes are not available in the dataset.

The Cz (Central Midline) region also exhibits strong transitions to the frontal areas, particularly FL and FR, indicating that central channels were also frequently selected. The TL and TR regions show notable transitions to frontal and central areas, which may be related to the memory and language functions of the temporal regions; this interpretation remains hypothetical.

Overall, frontal and temporal symbols dominate the transitions selected for Case 1. The observed transitions identify the channel pairs that the model relied on; they do not by themselves identify neural pathways affected in dementia.

The average transition table for Case 2, which focuses on the classification of dementia versus non-dementia, shows the most frequent transitions between DLob symbols. The FL region shows the highest transition frequencies, particularly to itself (11.75 times) and to FR (9 times), showing that frontal feature transitions were retained most often for distinguishing dementia. This pattern may be compatible with the frontal involvement reported in dementia, but it does not establish impaired frontal function.

The Cz region also exhibits high transition frequencies, especially to FL and FR, indicating that central channels were also frequently selected. Similarly, regions TL and TR show significant transitions to FL and FR, showing frequent temporal–frontal feature transitions. The transitions from TL to FL (10.75 times) and TR to FL (7.25 times) are frequent; whether they relate to the memory and language deficits of dementia cannot be determined from these data.

Table 13 shows that frontal, central, and temporal symbols were the most frequent in the features selected for differentiating dementia from non-dementia. This distribution is a property of the selected features and should be tested against established connectivity measures before a pathophysiological interpretation is made.

The FL and FR regions exhibit the highest transition frequencies, particularly within themselves and with each other. For instance, FL transitions to itself 14.25 times and to FR 12.25 times. Similarly, FR shows a high self-transition (12.75) and a strong connection with FL (11.75). Frontal feature transitions were therefore retained most often for differentiating AD from FD; the participant-independent accuracy for this case (57.60%) indicates that these transitions do not separate the two disorders reliably in unseen participants. The TL and TR regions also show strong transitions, particularly to the frontal regions. TL transitions to FL 10.75 times and to FR 8.5 times, while TR transitions to FL 8.75 times and to FR 8.75 times. Temporal channels were therefore also frequently selected in this task. The Cz region shows moderate transition values, particularly with FL (six times) and FR (4.25 times). The OL and OR regions display lower transition frequencies overall, with notable transitions to frontal regions, especially FL and FR. Occipital channels therefore contributed less to the selected features for this task. The PL and PR regions exhibit moderate transition frequencies, particularly with FL and FR. Table 14 shows that frontal and temporal symbols dominate the feature transitions selected for distinguishing AD from FD, with smaller contributions from central and parietal symbols. Whether these patterns relate to the different neuropathology of AD and FD requires confirmation with physiological connectivity measures.

The frontal regions, particularly FL and FR, exhibit high transition frequencies, especially within themselves and with each other. FL transitions to itself 11.75 times and to FR 7.75 times, while FR transitions to FL 9.25 times and to itself 6.25 times. Frontal feature transitions were therefore the most frequent in AD detection. The Cz region shows strong transitions, particularly to FL (five times) and FR (6.75 times). Both TL (Temporal Left) and TR show significant transitions, especially towards the frontal regions. TL transitions to FL 8.75 times, and TR transitions to FR 6.25 times. Temporal channels, which overlie regions associated with memory and language, were therefore frequently selected; this observation does not by itself demonstrate temporal dysfunction. The occipital regions, particularly OR, show moderate transitions, with OR transitioning to FR five times. Occipital symbols were less frequent than frontal and temporal symbols. The parietal regions (PL and PR) show lower transition frequencies overall, though there is some notable interaction with frontal regions. Parietal channels therefore contributed less to the selected features. The overall pattern indicates that the features selected for AD detection mainly involve frontal, central, and temporal channels. Table 15 summarizes the channel transitions used by the model for AD detection. Because the dataset mainly includes moderate-stage AD and the participant-independent accuracy was 63.48%, these patterns cannot be used to infer early AD detection or diagnostic or therapeutic targets.

The transitions involving the frontal lobes (FL and FR) are particularly high, with FL showing the most significant transition to itself (nine times) and FR (7.5 times). This frontal predominance may be compatible with the frontal involvement in frontotemporal dementia, but it is a feature-level observation.

There are notable transitions from FL and FR to the temporal lobes (TL and TR). For instance, TL transitions to FL 9.25 times, indicating frequent frontal–temporal feature transitions in this task. A relation to the language and memory impairments of frontotemporal dementia is possible but cannot be established without subtype information and connectivity measures.

The central midline region (Cz) also shows significant transitions to the frontal lobes (4.5 times to FL and 3.5 times to FR).

The transitions involving the occipital (OL, OR) and parietal (PL, PR) regions are less prominent; the largest of these is PL to FL (5.25 times).

Table 16 shows that frontal and temporal symbols dominate the feature transitions selected for FD detection. Given the participant-independent accuracy of 57.91% for this case, these patterns are not suitable for diagnosis or treatment planning.

**Figure 5:**
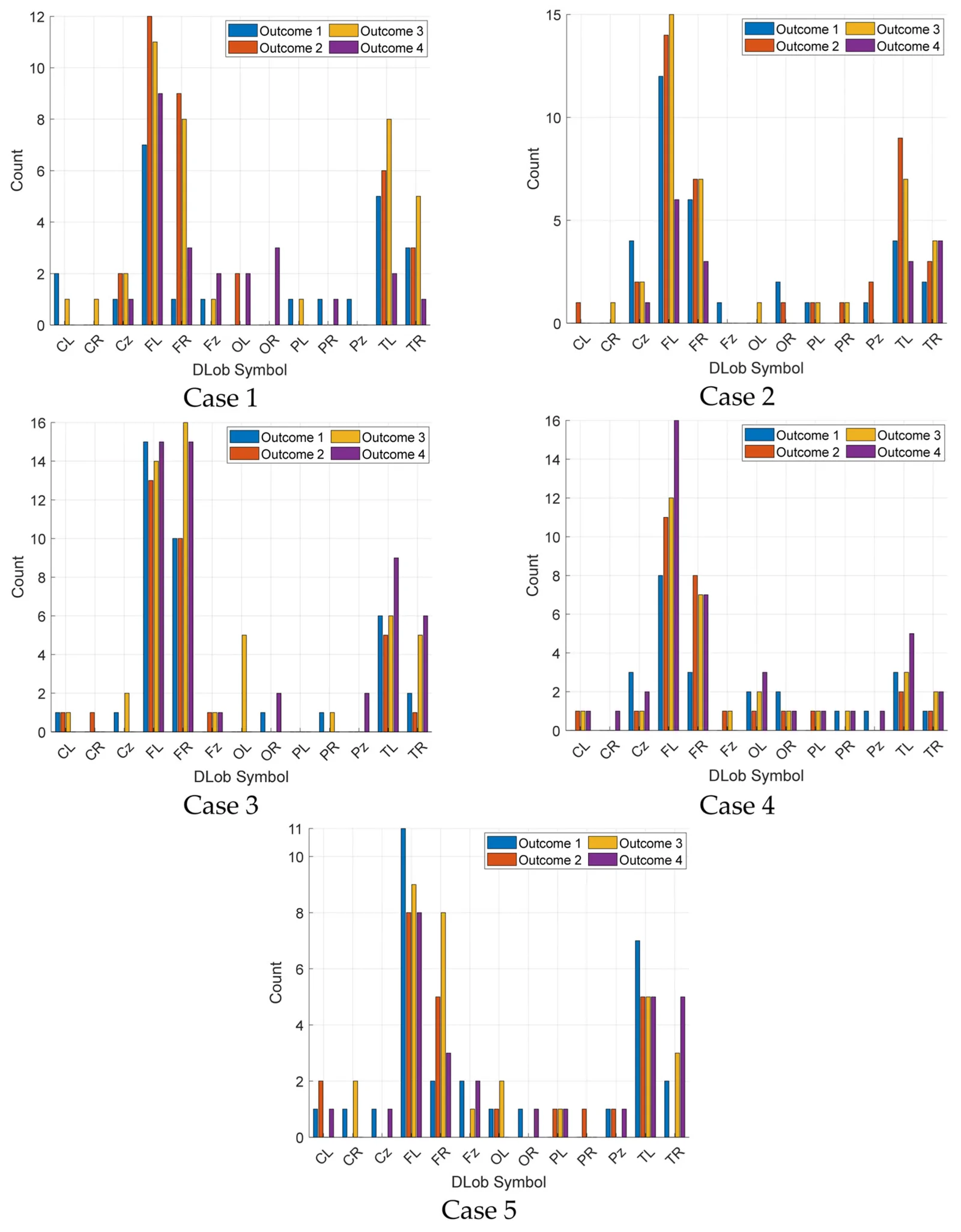
Histograms of the successive repetitions of DLob symbols.

Moreover, we have presented an analysis of successive repetitions of DLob symbols for the defined cases, and these results are demonstrated in Figure 5.

The successive repetition of certain DLob symbols, particularly FL and FR, across all cases indicates that frontal channels were the most frequently selected in all cases. Frontal involvement is expected in AD and in the behavioral and non-fluent variants of frontotemporal dementia, but subtype information is not available in this dataset. The temporal lobes (TL and TR) also show significant repetitive patterns, especially in cases involving FD, which may be compatible with temporal involvement in frontotemporal dementia (e.g., the semantic variant); this remains a hypothesis.

These histograms show that, in addition to symbol frequency, the successive repetitions of symbols differ between cases. Because the repetitions are derived from the ordering of the selected feature indices, they describe the feature representation and are not direct measures of neural activity. The differences between cases have not been tested statistically.

The findings, advantages, limitations and future work of this research are as follows:

**Findings:**

- Under segment-wise 10-fold cross-validation, which allowed segments from the same participant in the training and test folds, the PNPat and DLob-based SOXFE model achieved over 99% accuracy in all cases.
- The best segment-wise classification accuracies were 99.12% for Case 1 (AD vs. FD vs. Healthy), 99.23% for Case 2 (Dementia vs. Healthy), 99.74% for Case 3 (AD vs. FD), 99.30% for Case 4 (AD vs. Healthy), and 99.56% for Case 5 (FD vs. Healthy).
- Under participant-independent validation, the accuracies decreased to 51.19–63.81% (LOSO, segment level), 53.41–67.05% (LOSO, participant level), and 49.00–62.50% (nested participant-grouped 10-fold), which are the relevant estimates for unseen participants.
- The voted outcomes provided the best results in four out of five cases, under the segment-wise protocol.
- DLob sentences map selected channel transitions to scalp-region symbols. Their interpretation is restricted to the feature representation.
- The entropy values of the DLob sentences varied little between cases (2.7962–2.8924 bits) and describe the diversity of the selected feature symbols, not the complexity of cortical activity.
- Frontal symbols were frequently represented among the selected feature indices. This frequency is a property of the algorithm-derived representation and does not establish abnormal frontal activity.
- The frontal lobes are heavily involved in executive functions, decision-making, and behavior regulation, which are often affected in both AD and FD. The frequent selection of frontal channels may be compatible with this role, but it does not demonstrate frontal dysfunction. In particular, behavioral changes and personality alterations are prominent in FD, which is directly related to frontal lobe dysfunction.
- Temporal symbols were also represented in the selected transitions. Their diagnostic specificity was not established by the participant-independent results.
- Temporal regions are especially crucial in AD, as the temporal lobes are deeply involved in memory processing and language functions. The frequent selection of temporal channels in distinguishing AD from healthy controls may be compatible with this, but it is a feature-level observation.
- The Cz and Pz regions show varying levels of repetition, with their involvement being more prominent in Cases 3 (AD vs. FD) and 4 (AD vs. Healthy).
- The parietal lobes, associated with spatial processing and navigation, were more frequently selected in the FD-related cases than in the AD-related cases, especially in distinguishing FD from healthy subjects. The clinical meaning of this difference is uncertain.
- The generated connectome-style diagrams visualize the channel transitions selected by the model. They are feature-driven visualizations; their weak correlation with coherence- and wPLI-based connectivity (ρ = 0.15) shows that they should not be interpreted as neural connectivity.
- Frontal and temporal symbols dominated the selected transitions in all cases; their relation to the executive, memory, and language impairments of dementia remains to be tested.

**Advantages:**

- The feature extraction stage is computationally inexpensive at the recorded setting. The complete training cost remains dominated by supervised feature selection. Computational feasibility alone does not establish diagnostic utility.
- One of the most significant advantages of the proposed model is its ability to provide explainable results. By integrating the DLob symbolic language, the model generates interpretable outcomes that link each selected feature to a pair of scalp regions.
- The generated diagrams provide a transparent view of the channel transitions used by the model, which helps researchers to inspect and question the model’s decisions.
- BCT and PNPat provide a deterministic transition representation that can be linked to the selected channel-pair indices. Disease specificity and an accuracy advantage over non-symbolic features were not established.
- CWINCA reduced feature dimensionality by 34.25% on average in the original segment-wise analysis. Feature-set overlap across participant-grouped folds is reported separately in Table 11. Dimensionality reduction alone does not establish disease relevance.
- The tkNN configuration can be selected within participant-grouped training folds. Its relative component performance under the segment-wise protocol does not establish clinical generalization.
- This study provides an explainable symbolic feature engineering framework and documents, on a public dataset, how strongly the reported accuracy depends on the validation protocol.

**Limitations:**

- The dataset includes only 88 participants (36 AD, 23 FD, and 29 healthy) from a single center. Although 4661 segments were analyzed, segments from the same participant are correlated, so the effective sample size is close to the number of participants. The confidence intervals based on segment counts therefore underestimate the uncertainty, and the participant-level estimates have wide confidence intervals (e.g., 43.06–63.47% for Case 1).
- Participant-independent discrimination was insufficient for clinical use. In the three-class LOSO participant analysis, only five of the 23 FD participants were correctly identified; the segment-level FD sensitivity was 27.99%. The majority-class comparison also exposed limited accuracy beyond the cohort distribution. These failures restrict any diagnostic interpretation.

In the nested implementation, min–max normalization and supervised ranking were fitted once on each outer training set and shared by its inner splits. Inner selection scores could therefore be optimistic, although the outer-test participants remained excluded. Complete nesting, which refits both steps within every inner training split, was not performed and is required in future evaluations.

DLob stability was task-dependent, and Case 3 did not exceed the random-subset overlap reference. Non-symbolic features achieved higher nested segment accuracies in all tasks. Symbolic outputs therefore support feature traceability only. Their disease specificity, physiological interpretation, and reproducibility require independent validation.

- The cohort mainly includes moderate-stage AD (mean MMSE 17.75) and FD with heterogeneous severity; very mild, prodromal, or preclinical cases are not represented, so the usefulness of the model for early dementia detection has not been shown. The diagnostic labels are clinical; biomarker confirmation and FD subtypes are not available.
- Demographic and recording-related factors may act as confounders. The FD group was younger (63.6 ± 8.2 years) than the AD (66.4 ± 7.9 years) and healthy (67.9 ± 5.4 years) groups, the sex distribution differed between groups (female/male: AD 24/12, FD 9/14, healthy 11/18), and the recording length differed between participants. These factors were not controlled in the analysis.
- No independent external cohort was available. The results must be validated on independent datasets before any clinical claim can be made.
- DLob transitions and symbol entropies describe the selected feature representation. The transition diagrams had only a weak association with coherence- and wPLI-based connectivity (ρ = 0.15). This comparison does not validate a physiological interpretation of the DLob outputs.

**Future works:**

- We plan to test the PNPat and DLob-based SOXFE model on independent, multi-center EEG datasets with participant-independent validation.
- To improve participant-independent accuracy, participant-level normalization, domain adaptation, and features that are less sensitive to individual signatures will be investigated.
- XAI results of other brain-related disorders, such as epilepsy, Parkinson’s disease, and schizophrenia, will be extracted, deploying the presented SOXFE model.
- More SOXFE models will be presented.
- Clinical tools will be considered only after acceptable participant-independent performance has been demonstrated in external cohorts.
- Researchers will also focus on applying the SOXFE model to longitudinal EEG data to study the progression of dementia over time.

## 6. Conclusions

A symbolic EEG feature engineering framework was developed with BCT and PNPat. DLob maps selected transition indices to interpretable scalp-region symbols. The findings support the feasibility of algorithm-derived feature representations. They do not establish reproducible disease-related EEG characteristics.

Participant separation substantially reduced classification performance. Nested pooled segment accuracies were 49.00%, 62.50%, 55.99%, 61.51%, and 56.51% for Cases 1–5. Comparisons with the majority-class baseline and non-symbolic features did not establish a general accuracy advantage. These results do not support clinical deployment.

DLob sentences and diagrams provide feature-level traceability. Their physiological meaning and disease specificity remain unvalidated. The stability analysis was not uniformly above the random-subset reference, and the non-symbolic comparison did not support an accuracy benefit from symbolic features.

Future work should validate the approach in independent cohorts, improve participant-independent performance, and compare the DLob outputs with established connectivity measures before any diagnostic application is considered.

## Figures and Tables

**Table 1:** Demographic, clinical, and recording properties of the EEG dementia dataset [27].

No	Class	Participant (Number)	Sex (F/M)	Age, Mean (SD)	MMSE, Mean (SD)	Recording Time (min)	EEG Segment (Number)
1	AD	36	24/12	66.4 (7.9)	17.75 (4.50)	485.5	1943
2	FD	23	9/14	63.6 (8.2)	22.17 (8.22)	276.5	1104
3	Healthy	29	11/18	67.9 (5.4)	30	402.0	1614
Overall/Total		88	44/44	66.2	22.94	1164.0	4661

**Table 2:** Meaning of the DLob symbols used.

No	Symbol	Meaning
1	CL	CL denotes the left central region (C3 electrode), which overlies the left sensorimotor cortex and is mainly related to motor control and somatosensory processing. Central scalp electrodes do not record cerebellar activity.
2	CR	CR denotes the right central region (C4 electrode), which overlies the right sensorimotor cortex and has the same motor and somatosensory roles as CL.
3	Cz	This symbol refers to the central midline of the brain, particularly involved in motor control and sensory processing, especially movements that engage both hemispheres.
4	FL	FL corresponds to the left frontal lobe, a region crucial for higher cognitive processes like decision-making, logical reasoning, and problem-solving.
5	FR	FR stands for the right frontal lobe, which plays a significant role in emotional regulation, social behaviors, and spatial awareness.
6	Fz	This represents the midline of the frontal region, associated with executive functions, focused attention, and planning of movements.
7	OL	OL signifies the left occipital lobe, primarily involved in processing visual information, particularly related to the right visual field.
8	OR	OR is the right occipital lobe’s symbol, crucial for visual processing, especially concerning the left visual field.
9	PL	PL represents the left parietal lobe, involved in spatial orientation, sensory processing, and aspects of language comprehension.
10	PR	PR defines the right parietal lobe and is associated with processing sensory information and spatial orientation, and it plays a crucial role in integrating sensory data from different parts of the body.
11	Pz	Pz denotes the midline of the parietal region, essential for integrating sensory information from different body parts.
12	TL	TL stands for the left temporal lobe, important for processing language, auditory perception, and memory storage.
13	TR	TR symbolizes the right temporal lobe, involved in auditory processing, memory recall, and emotional responses.

**Table 3:** Segment-wise 10-fold cross-validation results (%) without participant grouping.

Case	Classifier-Based Results			Voted Results		
	Out	Acc.	GM.	Combination	Acc.	GM.
1	1	98.73	98.72	1-2-3	**99.12**	**99.09**
	2	98.88	98.86	1-3-4	99.06	99.00
	3	98.97	98.96	2-3-4	99.10	99.04
	4	98.63	98.59	1-2-3-4	99.12	99.08
2	1	99.06	98.76	1-2-3	99.16	98.98
	2	98.99	98.79	1-3-4	**99.23**	**99.01**
	3	98.97	98.80	2-3-4	99.16	98.99
	4	98.93	98.73	1-2-3-4	99.21	98.94
3	1	99.11	98.99	1-2-3	99.54	99.42
	2	99.47	99.33	1-3-4	99.64	99.52
	3	99.57	99.53	2-3-4	**99.74**	**99.72**
	4	99.72	99.72	1-2-3-4	99.57	99.41
4	1	99.02	99.02	1-2-3	99.18	99.19
	2	99.04	99.04	1-3-4	99.30	99.30
	3	99.13	99.15	2-3-4	**99.30**	**99.32**
	4	99.13	99.12	1-2-3-4	99.30	99.29
5	1	99.34	99.21	1-2-3	99.56	99.48
	2	99.48	99.41	1-3-4	99.56	99.48
	3	**99.56**	**99.56**	2-3-4	99.56	99.48
	4	99.52	99.47	1-2-3-4	99.56	99.48

Bold font face shows the best results.

**Table 4:** Performance (%) of the SOXFE model under segment-wise and participant-independent validation protocols.

Case	Validation Protocol	Accuracy (95% CI)	GM	BA	Macro-F1
Case 1 (AD vs. FD vs. HC)	Segment-wise 10-fold CV	99.12 (98.81–99.35)	99.09	99.09	99.08
	LOSO, segment level	51.19 (49.76–52.62)	45.43	48.01	47.96
	LOSO, participant level	53.41 (43.06–63.47)	44.56	49.93	49.13
	Nested, pooled segment level	49.00 (47.57–50.44)	43.50	45.96	45.90
	Nested, participant level	51.14 (40.87–61.31)	43.08	47.86	47.16
	Majority-class baseline, participant level	40.91	–	33.33	–
	Majority-class baseline, segment level	41.69	–	33.33	–
Case 2 (Dementia vs. HC)	Segment-wise 10-fold CV	99.23 (98.93–99.44)	99.01	99.02	99.14
	LOSO, segment level	63.81 (62.42–65.17)	56.58	58.84	59.00
	LOSO, participant level	67.05 (56.69–75.97)	57.41	60.52	60.85
	Nested, pooled segment level	62.50 (61.10–63.88)	55.42	57.64	57.75
	Nested, participant level	65.91 (55.53–74.96)	54.97	58.80	59.06
	Majority-class baseline, participant level	67.05	–	50.00	–
	Majority-class baseline, segment level	65.37	–	50.00	–
Case 3 (AD vs. FD)	Segment-wise 10-fold CV	99.74 (99.48–99.87)	99.72	99.72	99.72
	LOSO, segment level	57.60 (55.83–59.34)	51.70	53.65	53.67
	LOSO, participant level	57.63 (44.93–69.39)	50.12	53.50	53.28
	Nested, pooled segment level	55.99 (54.22–57.74)	50.26	52.15	52.16
	Nested, participant level	55.93 (43.29–67.85)	46.88	51.33	50.83
	Majority-class baseline, participant level	61.02	–	50.00	–
	Majority-class baseline, segment level	63.77	–	50.00	–
Case 4 (AD vs. HC)	Segment-wise 10-fold CV	99.30 (98.96–99.52)	99.30	99.30	99.29
	LOSO, segment level	63.48 (61.88–65.05)	63.17	63.23	63.21
	LOSO, participant level	66.15 (54.04–76.47)	65.65	65.76	65.76
	Nested, pooled segment level	61.51 (59.90–63.10)	61.21	61.27	61.24
	Nested, participant level	64.62 (52.48–75.12)	64.33	64.37	64.31
	Majority-class baseline, participant level	55.38	–	50.00	–
	Majority-class baseline, segment level	54.62	–	50.00	–
Case 5 (FD vs. HC)	Segment-wise 10-fold CV	99.56 (99.23–99.75)	99.48	99.49	99.54
	LOSO, segment level	57.91 (56.04–59.75)	54.16	55.57	55.58
	LOSO, participant level	57.69 (44.19–70.13)	53.23	55.77	55.31
	Nested, pooled segment level	56.51 (54.64–58.37)	52.87	54.24	54.24
	Nested, participant level	55.77 (42.34–68.40)	50.19	53.60	52.82
	Majority-class baseline, participant level	55.77	–	50.00	–
	Majority-class baseline, segment level	59.38	–	50.00	–

CI: Wilson 95% confidence interval, based on segment counts for segment-level rows and participant counts for participant-level rows. Nested segment metrics pool the outer-test predictions; participant metrics use majority voting. The majority-class baseline always assigns the most frequent class at the corresponding level. GM: geometric mean; BA: balanced accuracy; Macro-F1: macro-averaged F1-score. –: not available for that result in the reported analyses.

**Table 5:** Paired comparison of SOXFE with the majority-class baseline at the participant level.

Case	Protocol	b	c	Exact p	ΔAccuracy (95% CI), pp	ΔBA (95% CI), pp
1	LOSO	22	11	0.080	12.50 (0.00–25.00)	16.60 (6.92–26.29)
1	Nested	21	12	0.163	10.23 (−2.27–22.73)	14.53 (4.66–24.50)
2	LOSO	12	12	1.000	0.00 (−10.23–11.36)	10.52 (0.37–21.08)
2	Nested	11	12	1.000	−1.14 (−11.36–9.09)	8.80 (−1.44–19.07)
3	LOSO	8	10	0.815	−3.39 (−16.99–10.17)	3.50 (−8.74–16.11)
3	Nested	7	10	0.629	−5.08 (−18.64–8.47)	1.33 (−10.75–13.37)
4	LOSO	18	11	0.265	10.77 (−4.65–26.15)	15.76 (3.85–27.12)
4	Nested	18	12	0.362	9.23 (−7.69–26.15)	14.37 (1.99–26.19)
5	LOSO	9	8	1.000	1.92 (−13.46–17.31)	5.77 (−7.20–18.79)
5	Nested	8	8	1.000	0.00 (−15.38–15.38)	3.60 (−8.64–16.44)

b: SOXFE correct and baseline wrong; c: SOXFE wrong and baseline correct. p: two-sided exact McNemar test. Differences and confidence intervals use paired participant outcomes; bootstrap: 10,000 participant resamples, seed 1. pp = percentage points.

**Table 6:** Class-wise diagnostic metrics (%) under segment-wise, LOSO, and nested validation.

Case	Positive Class	Validation	Sensitivity	Specificity	Precision	NPV	F1-Score
Case 1 (AD vs. FD vs. HC)	AD	Segment-wise 10-fold CV	99.33	99.45	99.23	–	99.28
		LOSO, segment level	62.02	63.36	54.75	–	58.16
		LOSO, participant level	69.44	61.54	55.56	–	61.73
		Nested, segment level	59.34	62.10	52.82	–	55.89
	FD	Segment-wise 10-fold CV	99.00	99.58	98.65	–	98.82
		LOSO, segment level	27.99	86.48	39.11	–	32.63
		LOSO, participant level	21.74	86.15	35.71	–	27.03
		Nested, segment level	26.81	85.66	36.72	–	30.99
	HC	Segment-wise 10-fold CV	98.95	99.64	99.32	–	99.13
		LOSO, segment level	54.03	73.81	52.22	–	53.11
		LOSO, participant level	58.62	79.66	58.62	–	58.62
		Nested, segment level	51.73	72.53	49.94	–	50.82
Case 2 (Dementia vs. HC)	Dementia	Segment-wise 10-fold CV	99.70	98.33	99.12	99.44	99.41
		LOSO, segment level	74.99	42.69	71.18	47.48	73.04
		LOSO, participant level	79.66	41.38	73.44	50.00	76.42
		Nested, segment level	73.45	41.82	70.44	45.49	71.92
Case 3 (AD vs. FD)	AD	Segment-wise 10-fold CV	99.79	99.64	99.79	99.64	99.79
		LOSO, segment level	67.99	39.31	66.35	41.10	67.16
		LOSO, participant level	72.22	34.78	63.41	44.44	67.53
		Nested, segment level	66.08	38.22	65.31	39.04	65.69
Case 4 (AD vs. HC)	AD	Segment-wise 10-fold CV	99.28	99.32	99.43	99.13	99.36
		LOSO, segment level	65.98	60.47	66.77	59.62	66.37
		LOSO, participant level	69.44	62.07	69.44	62.07	69.44
		Nested, segment level	63.92	58.61	65.03	57.44	64.47
Case 5 (FD vs. HC)	FD	Segment-wise 10-fold CV	99.09	99.88	99.82	99.38	99.45
		LOSO, segment level	43.12	68.03	47.98	63.62	45.42
		LOSO, participant level	39.13	72.41	52.94	60.00	45.00
		Nested, segment level	42.12	66.36	46.13	62.63	44.03

For Case 1, metrics are calculated one-versus-rest for each class. NPV: negative predictive value; –: not applicable.

**Table 7:** Confusion matrices (rows: true class; values: number of segments or participants assigned to each predicted class in the order given).

Case	True Class	Predicted Order	Segment-Wise 10-Fold CV	LOSO, Segment Level	LOSO, Participant Level	Nested, Segment Level	Nested, Participant Level
Case 1 (AD vs. FD vs. HC)	AD	AD/FD/HC	1930/6/7	1205/258/480	25/6/5	1153/276/514	24/6/6
	FD		7/1093/4	477/309/318	11/5/7	485/296/323	11/5/7
	HC		8/9/1597	519/223/872	9/3/17	545/234/835	9/4/16
Case 2 (Dementia vs. HC)	Dementia	Dementia/HC	3038/9	2285/762	47/12	2238/809	47/12
	HC		27/1587	925/689	17/12	939/675	18/11
Case 3 (AD vs. FD)	AD	AD/FD	1939/4	1321/622	26/10	1284/659	26/10
	FD		4/1100	670/434	15/8	682/422	16/7
Case 4 (AD vs. HC)	AD	AD/HC	1929/14	1282/661	25/11	1242/701	24/12
	HC		11/1603	638/976	11/18	668/946	11/18
Case 5 (FD vs. HC)	FD	FD/HC	1094/10	476/628	9/14	465/639	8/15
	HC		2/1612	516/1098	8/21	543/1071	8/21

**Table 8:** Number of features selected by CWINCA from the 361 features of each feature vector and the mean reduction ratio.

Case	Out 1	Out 2	Out 3	Out 4	Mean	Reduction (%)
1	189	237	218	230	218.50	39.47
2	270	261	238	205	243.50	32.55
3	245	239	314	329	281.75	21.95
4	168	212	217	268	216.25	40.10
5	218	204	268	217	226.75	37.19
All					237.35	34.25

**Table 9:** Information entropies of the generated DLob sentences.

Case	Out				Mean
	1	2	3	4	
1	2.8550	2.8407	2.8561	2.8417	2.8484
2	2.8589	2.8453	2.8365	2.8645	2.8513
3	2.8906	2.8913	2.8964	2.8915	2.8924
4	2.7820	2.7920	2.7832	2.8277	2.7962
5	2.8748	2.8540	2.8780	2.8588	2.8664

**Table 10:** Studies using the same EEG dementia dataset and their validation strategies. Accuracies obtained with different validation strategies are not directly comparable.

Study	Methods	XAI	Number of Subjects	Class	Split Ratio	Participant-Independent	Accuracy (%)
[32]	N-BodyPat	Yes	36 AD, 23 FD, 29 CN	3	10-fold CV	No (segment-wise)	99.64
[33]	Azimuthal equidistant projection technique	No	36 AD, 29 CN	2	10-fold CV	Not specified	97.00
[34]	Convolved connectivity Features	No	36 AD, 23 FD, 29 CN	3	Leave-one-subject-out CV	Yes	84.85
[35]	Dual input convolutional encoder	No	36 AD, 29 CN	2	Leave-one-subject-out CV	Yes	83.28
[36]	Convolutional neural network, long short-term memory	No	36 AD, 23 FD, 29 CN	3	80:20	Not specified	97.40
[37]	Spatiotemporal EEG analysis	No	36 AD, 23 FD, 29 CN	3	60:20:20	Not specified	84.59
[38]	Forward–backward Fourier transform	No	10 AD, 8 CN	2	80:20	Not specified	95.61
[39]	Entropy analysis, random forest	No	36 AD, 29 CN	2	Leave-one-person-out CV	Yes	95.65
[40]	Statistical and entropy analysis	No	36 AD + 23 FD, 29 CN	2	10-fold CV	Not specified	93.50
[41]	Vector quantized variational autoencoders	No	36 AD, 23 FD, 29 CN	3	5-fold CV	Not specified	93.30
[42]	Fast Fourier transform	No	36 AD, 23 FD, 29 CN	3	85:15	Not specified	98.53
[43]	Time-frequency functional connectivity alterations	No	36 AD + 23 FD, 29 CN	2	Unspecified	Not specified	89.77
[44]	Support vector machine	No	36 AD, 23 FD	2	Leave-one-out CV	Not specified	96.60
[45]	Multi-threshold recurrence rate plots	No	36 AD + 23 FD, 29 CN	2	Leave-one-out CV	Not specified	86.36
[46]	Convolutional neural network	No	36 AD, 23 FD, 29 CN	3	60:20:20	Not specified	93.14
[15]	Fractal dimension, long-range temporal correlations, band power	No	36 AD, 23 FD, 29 CN	3	Group comparison (no classifier)	Not applicable	Not applicable
[16]	Prefrontal spectral, aperiodic, and processed EEG indices	No	35 AD, 23 FD, 27 CN	2	Group comparison (no classifier)	Not applicable	Not applicable
[17]	Dynamic connectivity, convolutional neural network	Yes	36 AD, 23 FD, 29 CN	3	Subject-wise 5-fold CV	Yes	93.60
[18]	Functional connectivity, stacked FgMDM ensemble	No	36 AD, 29 CN	2	Leave-one-subject-out CV	Yes	73.85
[19]	Copula connectivity, dynamic-threshold graph convolutional network	No	36 AD, 23 FD, 29 CN	3	Subject-level stratified 5-fold CV	Yes	82.00
[20]	Dynamic mode decomposition, clustered pattern projection, linear SVM	No	36 AD, 29 CN	2	Nested leave-one-subject-out CV	Yes	76.70
[32] (re-run)	N-BodyPat	Yes	36 AD, 23 FD, 29 CN	3	Segment-wise 10-fold CV	No (segment-wise)	97.80
[32] (re-run)	N-BodyPat	Yes	36 AD, 23 FD, 29 CN	3	Leave-one-subject-out CV	Yes	52.00
Our study	PNPat and BCT-based SOXFE	Yes	36 AD, 23 FD, 29 CN	3	Segment-wise 10-fold CV	No (segment-wise)	99.12
Our study	PNPat and BCT-based SOXFE	Yes	36 AD, 23 FD, 29 CN	3	Leave-one-subject-out CV	Yes	51.19
Our study	PNPat and BCT-based SOXFE	Yes	36 AD, 23 FD, 29 CN	3	Nested participant-grouped 10-fold CV	Yes	49.00

CN: cognitively normal (healthy) controls. Participant-independent: Yes, if all segments of a participant were confined to either the training or the test set; Not specified, if this was not reported. The [32] (re-run) rows were obtained in this study by evaluating N-BodyPat under the same validation protocols as the proposed model (Case 1). For [18] and [20], the AD versus CN accuracy is given; [15] and [16] reported group comparisons without a held-out classifier.

**Table 11:** DLob stability and non-symbolic feature comparison under nested validation.

Case	Mean Jaccard	Random-Subset Jaccard	Selected Features/Fold	Transition ρ	Non-Symbolic Accuracy (%)	Non-Symbolic BA (%)
1	0.64	0.42	214	0.74	50.50	46.96
2	0.67	0.49	239	0.76	63.00	58.44
3	0.61	0.62	277	0.70	57.00	53.35
4	0.66	0.42	212	0.73	64.50	63.77
5	0.63	0.44	222	0.72	58.00	55.74

Jaccard: Average pairwise overlap of fold-wise CWINCA feature sets. The random-subset reference is the expected Jaccard index of two random k-feature subsets of the 361 features, k/(2·361 − k), with k equal to the mean number of selected features per fold. ρ: Spearman correlation between fold-wise and full-data average DLob transition tables. Non-symbolic comparator: Band-power and statistical channel features with the same CWINCA–tkNN–CMV pipeline.

**Table 12:** Average transition table of Case 1 (classification of AD vs. FD vs. Healthy).

		DLob Symbol (to)												
		CL	CR	Cz	FL	FR	Fz	OL	OR	PL	PR	Pz	TL	TR
DLob symbol (from)	CL	1	1.5	1.25	3.25	4.5	2	1.5	1.75	1	2	2	3.75	2
	CR	2	0.5	0.75	3.25	2.25	1.25	1	0.75	0.5	0.5	1.25	1.75	2.25
	Cz	1.25	1.25	1.5	5	4	0.75	2.25	2	1.5	1.75	2.5	4	2.25
	FL	4	2.75	5	10.25	7.5	3.25	2.75	5	3.25	4	3	9.5	6.25
	FR	3.75	2	4.75	8.5	6.75	3.25	4.75	3	2.75	1.5	3.25	7	5.75
	Fz	1.75	1.5	1.75	4.25	3	1	1.5	1.5	0.75	1.25	1.5	3	1.5
	OL	1	0.75	1.5	4	3.5	1	1.25	1.5	0.75	1.5	1.5	2.75	2
	OR	1.5	1	2	3	4.75	2	1.25	1	1.5	2	1.5	3.25	3.5
	PL	1	0.75	1.25	3.75	2.25	1.75	0.25	2	0.5	1	1.5	1.75	2.5
	PR	1.5	1	2	3	1.75	1	1.25	1.5	1	0.75	2	2.5	2.25
	Pz	2	1	1.5	5	3	1	1	1.25	1.25	1.75	0.25	2.5	2.25
	TL	4.25	1.75	4.25	7.25	6	3.75	2.5	3.75	3	2	2	5.75	7.25
	TR	2.5	2.25	2.5	6	7.75	2	1.75	3.25	2.5	1.5	1.5	6	3

**Table 13:** Average transition table of Case 2 (classification of dementia or not).

		DLob Symbol (to)												
		CL	CR	Cz	FL	FR	Fz	OL	OR	PL	PR	Pz	TL	TR
DLob symbol (from)	CL	0.25	1.25	2	4.25	3.75	1.75	1.25	2.75	0.5	1.5	2	3.5	2.75
	CR	1.25	0.25	1.25	2.5	3.25	1.5	1	1	1.25	1	1.25	3	2.5
	Cz	2	1	2.25	5.25	5.5	1	1.75	1.75	1	2.5	2.25	4.75	2.75
	FL	3.25	3.75	6	11.75	9	6.5	3.75	3.5	4.75	4.5	3.5	8	7.75
	FR	4.25	2.75	4.75	10.75	5.75	3.25	4.5	4.5	3	2.75	2.75	8.25	6.5
	Fz	1.75	1.25	2	3	2.25	0.25	2	2.25	2.25	1	2.25	4.5	2.25
	OL	1	1.25	2.5	3.25	3.5	1.5	0.25	1.5	1.25	1.75	1	3.5	3
	OR	2.5	0.75	1	4.5	4.5	1.25	1.5	0.75	2	2	1.5	4.5	3.5
	PL	1.5	1	1.25	6	2.25	2	1	2	0.75	1	1.5	2.75	1.75
	PR	1.75	0.75	1.75	4	3	0.25	0.75	2.25	1.5	0.5	2	4	3.25
	Pz	2.75	1.25	1.75	3.25	2.75	2	1.75	1.5	1.25	1.5	0.75	2.5	1.5
	TL	3	2.75	4.25	10.75	10	3	3.25	3.75	2.75	2.75	2.75	5.75	5.5
	TR	2.25	3	3	7.25	8	2.75	2.5	2.75	2.5	3	1.25	4.75	3.25

**Table 14:** Average transition table of Case 3 (classification of AD vs. FD).

		DLob Symbol (to)												
		CL	CR	Cz	FL	FR	Fz	OL	OR	PL	PR	Pz	TL	TR
DLob symbol (from)	CL	0.75	1.75	2.25	4.75	5.25	2.5	1.25	2.25	1.25	1.75	2.5	3.5	2.5
	CR	1.50	0.25	1.75	4	4	1.25	1.5	1	1	1.5	1.5	3.5	4
	Cz	2	2.25	0.75	6	4.25	1.25	1.75	2.5	0.75	1.5	2.25	5.25	2.75
	FL	5.50	3.5	4.50	14.25	12.25	5.75	4	4.5	4	5	5.25	10.25	11.5
	FR	3.75	3.5	6.75	11.75	12.75	4	5.25	4	3.25	4.5	4.25	8.5	7.5
	Fz	2	1	2.25	5.75	4.50	0.75	1.75	1.75	1	0.75	2.25	4.5	3
	OL	1.50	2	1.5	4.75	5.75	1.25	1.25	1.5	1.25	1.5	1.25	3.75	2.5
	OR	2.25	1	1.75	4.25	3.75	2.75	1.75	0.75	1.5	1.5	2.25	4.75	4.5
	PL	1.75	1.5	1	4.25	3	1.5	1	1.5	0	2	1.5	2.75	2.5
	PR	1.50	2.25	2.25	5.25	3.5	0.75	1.75	2	1.75	0.5	1.5	2.75	2.25
	Pz	2	1	1.5	5.75	4.25	1.75	1.75	2.50	2.5	1.25	0.5	4.75	2
	TL	4.75	3.25	4	10.75	8.5	3.75	3.5	5	3	3.75	3.25	6.5	7
	TR	3	3.5	2.75	8.75	8.75	3.75	3.25	3.5	3.25	2.25	3.25	6.25	3.5

**Table 15:** Average transition table of Case 4 (classification of AD vs. Healthy).

		DLob Symbol (to)												
		CL	CR	Cz	FL	FR	Fz	OL	OR	PL	PR	Pz	TL	TR
DLob symbol (from)	CL	0.75	1.5	1.75	3.25	3.25	1.5	1	2.25	0	2	1.75	4	2.25
	CR	1	0.25	1	3	3.75	1.25	1.5	0.75	1	0.25	1.25	1.25	1.75
	Cz	1.75	1.5	1.75	5	6.75	1.5	1.25	2.5	1	1.5	1.25	3	2.75
	FL	4	2.75	4.25	11.75	7.75	4	4.25	3.25	3.25	2.75	1.25	5.75	7.5
	FR	3.25	3.25	4.5	9.25	6.25	2.25	3.5	4.5	2.5	2.75	3.5	7.5	5.75
	Fz	1.25	1	2.5	4.5	3.5	0.50	1.25	1.25	1.25	1.25	2.25	3.25	1.75
	OL	1.25	1.5	2	2	3.5	1.5	2	1.75	1.5	1.75	1.5	3.25	2.25
	OR	2.75	0.50	1.75	3.25	5	3	1.25	1.25	1.5	1.75	1.25	3.75	2.75
	PL	0.5	1.5	1.5	2.5	2.5	1.75	1.25	1.75	0.75	0.75	1.5	1.5	2.25
	PR	1.5	0.50	2.25	2.25	2.75	0.5	2.25	2	2.25	0.75	1.75	3.25	2.5
	Pz	1.75	1	2.25	1.5	3	1.75	1.25	1.75	1.75	1.5	0.5	2.5	2.25
	TL	4	1	2.5	8.75	4	3	3	4	1.25	4.5	3.5	3.25	4.75
	TR	1.5	1.75	3.5	6	6.25	2.75	2	2.75	2.25	3	1.5	5	1.5

**Table 16:** Average transition table of Case 5 (classification of FD vs. Healthy).

		DLob Symbol (to)												
		CL	CR	Cz	FL	FR	Fz	OL	OR	PL	PR	Pz	TL	TR
DLob symbol (from)	CL	1	2	2	4.75	4	1.75	1.25	2	1	0.75	1.75	2.75	3.75
	CR	1	0.75	1.5	3	2.25	1.75	0.75	0.50	1.25	1.25	0.5	3	3.25
	Cz	1.75	1	0.5	4.5	3.5	2.5	1	1.5	2	1.75	1.75	3.5	4
	FL	4.75	3.25	4.5	9	7.5	4.75	3.5	4.75	5	3	4.25	9	8.25
	FR	2.75	2.75	3.75	8	4.5	3.25	3	4	3.25	3.25	3	8	6.25
	Fz	0.75	1.75	2	5.5	3.25	1.25	1.25	1.75	1.5	0.5	1.75	4.75	2.25
	OL	1.25	1	1.5	2.5	3.5	1.25	1	0.75	0.5	0.75	2	2.25	1.25
	OR	1.75	0.25	1.25	4.25	4.25	1.5	0.75	0.50	1.5	1.75	1	3.25	3
	PL	1.25	1	1.5	5.25	2.25	1.25	0	1.5	0.75	1.5	1.5	2.5	3
	PR	1	1.25	1.75	4.25	2.75	0.50	1	1.5	0.5	0.25	2	2.25	2.25
	Pz	3	1	2	3.5	3	2	1.75	1.75	1.75	1.5	0.75	3	2.5
	TL	3.75	1.75	3.75	9.25	7.75	4	2.25	2.75	1.75	3	4	5.5	5
	TR	4.75	3	3.25	8.25	7.5	2.25	2	1.5	2.25	2	3	5	2.5

**Figure A1:**
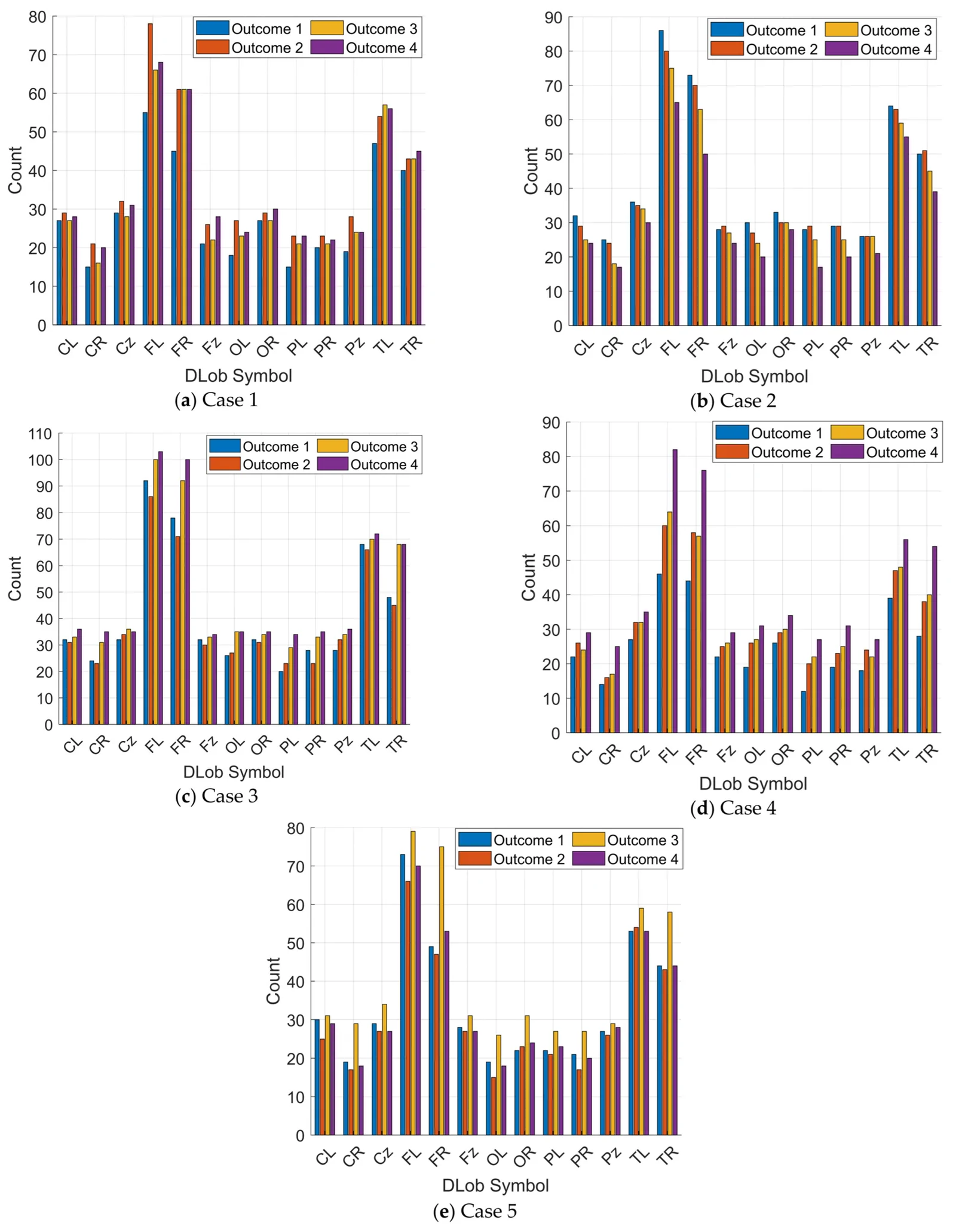
Histograms of the DLob symbols used.

## Data Availability

The EEG data analyzed in this study are publicly available in the OpenNeuro repository (dataset ds004504; https://openneuro.org/datasets/ds004504, access date 11 February 2026). The MATLAB implementation of the SOXFE model, including the scripts of the segment-wise, LOSO, and nested participant-grouped protocols and the fixed random seed used to generate the participant-wise fold assignments, is available at https://github.com/mhmtbygn/PNPat_SOXFE_Dementia, access date 11 February 2026.

## Appendix A

**Table A1:** The generated DLob strings for all cases.

Case	Out	DLob String
1	1	PLFLTRFzCzFRTLFLORFLTLORFRCLCRFzPRFLFRCzCzPzPLFLTLCzFLFRTRFROLFLFROLFLPRTLFRPzPRCzFzOLFLPRORFzFRPLFzFLORPRTLFLTLPRTLTLCzORTLFzFLFLTLFLTRFRCzFLFzFzFRCRFRTRFLFLCLPRPzCRFLTRFRCLFzTLORTLFzFLCzPzCzTLFRCLCzTRCLCLPzTLFLTRTRFLORTROLFLFLFRFLCRTRPLPzPzCLORTLTRFLCzTRPzORCzFLCLFROLPzCLPRPLORCzORFRFLFRTRTRCRCzTLCzPRCRFLPzTLTRPLTRTLORFzCLFLCzFLOLFRFzTLORFROLOROLTLFLTLFzPzTRPRFLCzFLFLFRFLTLFRFRPRFLFLFzCLFRCzTRORPLCzFRTLFLCRTLFLTLOLPzFzFRFLTLPzCLOLPRTRCRCzTLPLTRORFRCRTLCRPzFRFzOLFLFLCzFRORTRCRPLPLFLFzTRORFLORFRTLTRTLTLTRCzORPRTLCLFzTLTLOLCLTLTRFROLPRPRCzCRCLFRCLOLFRFLPLORCzTLCLOLFzFLTRFRFLTRFLTLTRORTRORCLCLFLOLTRTLFzTLPRPLFRFzORFLTRCRTRCzTLFRPzFRTRPzFRFLPROROLPzFLFROLCzCLTLTLTRTLFRTRFLTLPRCLORPzTRTLTLPzCLORPRTRTRFRCzPLFRPRCzFRCzCRFLFLTRCLTLFRCLTRCLFRPLFRCLCRCLPL
	2	FLTLCLFRORTLFzTLORCzTLFLPRCLTLFzCLTLFzFLCzTLORFLFLFLPRCzFLFRTLORPzCzTLPRCLTRFRCzCzPRFzTRFRCRFzPzFRTRFRFzFRCzCzFROLCLFRTLCLTLCLFLFLORTLFLFzCRORTLFRCLTLOLCzPzFLFzOLFzCLPzCzFLPzOLOLFRORPzTRFLTRFzCzFRTLTROLTLORTRFzCzPROLFLPLORFzPzTLTLFLFLCzFRPzFLOLTRTRFLTRTLFLCzORTLFzORPLFLPLTRPRPzPLFzPLPRPzCRFRFLFRFRFLTLPRCzFLFRORFRCLTRCRTRTLORPRTRORTROLORFLTRTLTLCLFRTROLTRFRORPRORFzORCRFRCLCRFLTLTRCzPzFzFLTLPLTLCzCLTRFLOROLPRTRFROLOLPROLTLCzTRPzCLPzTLTLFRFLOLFLPzCzFRTLFRFRTLCLCzCRFLPRTLCLORTLFLTLPzFLCRFRCzPzPRFRTRCzTLTRFRFLORPRFLFRPLORCLFzTLFLCRCLFzCRTLFLFLTLCLFLTLFLCzOLORTRFRPzFLTLCzPzORFLFRPzFRFRPzFLFLFLFRFLFRFLPRFLCRFRORFRTLTLPzFLFLPLCLORTRFROLTLTLFLFzCRCLFzCLTLTLTRCLCRTLTRFLCzPLPRCRTRFRTLTRFLPLFzPRFzOLPLTRCzCRTRPRFLFLFRTLPLCzPLFLPzFLPRFRFLTRTRCROLCzFLCLPRFRFRFRFLFRTLTRCLPRTLCzOLFROLFRFLTRFRTLFzFLOLFLFzFRFRFRFzFLORFRFRPLFRFLPRFLCLFLFLPzPLFzCzTRPLCRFLFLPLFLFLCzOLCRFLCLPLFROLFLPzCRCLFRTRPLPzTRFRFLPzFRFRTRTRFLCRPzFLORTRTLPLORPLPzTRPLCROLPLFLCzOLFROLFLCL
	3	FzCRORFLFLTLCLFLCLFRPzPRFzPLFLFLFRCRFLFRFLFRPRCzOLORFzTLFLFLFRFLCzFROLTLTLCLFzPzFRTLTLFLFzFLOLFzORPLCLTLOLFRCzPzTRFzPzFRPzOLFRTRORFzTRORFRCzCLPzTLPRFzTRORCzFLPLFLTRPzCzPRTRFLORFLTLORTLTLFLFLFzFLCRTLFLTLCzPzFLTLFzFRTLFRFzFRORCRFRCLCRCRFRFRCzFRTRTLFzFzTLFRTRTRTRFLFLFLOLPzCLOLCLFLFLTRCRPRORTRCzCzPRORTRCzTLTLORCLCzCzTLFLCzFLCROLTLCLFzPRFLTRTLPzFzFRORPROLCRTLCzPLFLPzPRCLFLTLCLTRCzFRFzORFLCzPLFLCLTLORTRTLFLTLPRCRFLFLPLORFLPzTLFLTRTRFRFzOLTLOLFRCLFRTLOLFRFzCzFLOLORPzOLTRFRPLPRPzFLPRFLORCLFLTROLFRPLTROLCzFLPRTLFRORCzCLPRFRTRFLPRFRTLFRFzFLFLTLCzFLFRTLFRPzORFRTLFRCRCLFLFLPLTLPLTRPLCzOLFLTLTRFROLTLORFRCzFLFRCzTRFLFRTLFRFRFLCzFRFLTRTLTLPLPzTRCLPRFLCLPRTRFRFROLTLTLTRFLOLPRCLFRORTLTLPzPRCRTRPRPzORPLTLPLFLPzFRFRFzFRTLTRPLPzTRFRPzFLCLFROLPzTLFRTRCzORFRFRFRFROLCRFzFLTLTRTRFRTRFLFRFRFLFLORCLCLORCRPLFRCLTLPLTRPRFLFRTLCzTLPLPLCLPzPLTLCRFLTLTRTLTLTRFRFLOROLCLTRTRPLTRTLCRPzFLCLCzTR
	4	CLOLFLFLCLPRCzCLFRCLTRFLFRCzFzFLOLPzFLORFROLCzTLCzFRTRFzFzFRCLCzORFLTLOLFLTLTRTRPLCzOROLCzORCzFRTRFRTRPRPzCzCRFzTRFRPRPzFLORORFRFLCzOLFLPLFLTLFzCRTRFzOLOLTLCLFRPzPRPLTRFLFLFRFLTLORTLOLOLPROLTRTLCLPzFzCRCLTLFLFLCLFLFLPLORFRFLPRTLFzTLTLFRFRPzFLFRTLFLPRCzTLFRFzTRCRFLORFRPLFzFLFzPRORTLFzCLPzFLTRCLPLORTLTRFRCzFzFzPzFRFLPLFLFzTLTRCLFRTRTLFLCRFRFLFLFLFLTRFLFLCzTLORTRTLCRCzCzOLTLCzFLTLOLFRPzTLTRTLCzFLPzCLFRFLFLTLOLFzCzTRCRPRFLPRPRTLTRFLCLTLTLCRCLFRCzPzTLCzPRTRFROLFLTLPLFLTLTRORFzTLFRPLFRCzPRPzFLCLTRORTRTLPzPRTRPzPLOLORFzCLTLFRFLPRFRCzOLFRFRCRPzFRFRTLTRPLTLTRFzORORCLFLCRFLFzFLCzPzORFRCRTROLTRTLFRTRTLPzTRORORFLORTRFLTRTLPLCLTLFLORCzTLCLORPzFRFLOLOLTRTLPRTLCLCLCRFRCzFLFzTLTLCzFLCzFLORPRTRCzTRFLFRORORFRFRTLFLTLCRFLCRTLFzFRTRORFRFRPLCRFzFRFRTLFRFLTLTLPLPzFLTRCLFLORPzCLFzPRCLPLFRTLCRPzTLCzFRFRPRFRTRFzPLCzPLFRPLTRCLOLFLFLPzOLFRTLTRFRPROLPLFzFLFLTRFRFRPLTLFRORPLPRPRPLTLFLFRCLFRFzFLCLPzFLTRPzCRTRFLPLFzCzFRCRCRORCROLTRFRFRFL
2	1	TLFLTRTRCRFzPLFLFRCLFRCzFLFRPRTLCzFRFLORORFLORTLFLFzORFzTRFzPzFzOLPzCLFRCRTRTROLFROLFLTLPRPzCLPzCRFRCRCLFRCzCzORPzCLOLTLOLFLTRFRFRPRTRFLPzPRFRCRPRTLCzPRPLCzTLFRTRCLTRTLFRCzPzORPRCzFLFLTLORTLPLPLFLFLTLORTROLFLPLORFRFLFzFRORFRPRORTLPRCzTLTLPzPzTLFLCLCzFLFzTLFLTLOLFRTLCRFRTRTLOLTRFLCzFRFLCzCzFzTRFROLTRFROLFRFLCLTLFzCLTLFLPLPzFzPzFRTLCzTRPRCLFLFLTRFRFRFLPRFLTRTLCLPRTLFROLORORPzCLCzCLORTRFLFzFLFRPzCzCLTLCzFRFLTRPLFLPLFLCRORCLTRCzCzTLTRPRTLFzFzFRCLTRCzPzFLFLFzOLTLFRTLFLPLFzTLFzPRTRCzFRFLTRFzTRTLORTLCzCzFLCRPzCLOLORFRPLTRFRTLTLCLFRCRFRORCLTLTRORCRFLFLCzTLCLFLTRFzORFLORFRFLPLFLTLFRCLFLTLOLCRCzOLFzPzCzFLFLPRTRTLTRCzOLCRTRPLFRFzTLOROLORFLFLCLFLPRTLPzORPRTRCzOLTRCRFLFLFLCLFRORTLFLCzFRTLFRORTLTRTLTLFRTRFRPLTLFLTRCLOLPRPzOLPzTLFLTRFRORPzFLFLPzFLFzFLOLFLTLFRPRORFRFRTLFLTRFLFzTRPzTLCRTLTRCzPLORCzCLCRFRFzCROLFzPLFLTLCRPLPzFRTLFLTLPLTLFRTLTLFzCzPLTRCLFLFLPRORPLOLCzTRPzFRFRFLFLCRPRFRPzOLFRTRFLCLPLFLFRFLPLORTRFLOLPRPLPRFROLFRTLPRCzFLORCRCLFzPLCRTRFRTRTLCRFLOLCLOLPLCzTRPLPRCLFRFLPLFLFROLCRTRTLPRFLPzFLFzPRTRFRCLFLFRCRFRFLPRFLFRFRPLTLFRTLPLFRCRORFRFLPRFRFRTRFLCLFLFROLFLFRFzCzCRTLFLFRFL
	2	FRFLFLORFRTRORTLORCzCzFLFzFLFLTRORTRFLFRTLFzCzFRFLTLCzCLFzCRTLPRORFLFLFRORCLPzORTLFLFzORPRFRPzOLCzTLCzFRTLFRFRCzTRTRFLOLTLFLTRCRFzCzORPLCLTRCzFLOLFRCLFRCLFzTLFLFRORFRTLFRCzCzPLFRCLPzTLFLFLFzTLTLTLFRFzTLFzTRFzFLFLCzPzPzCLTRCLCLCzPRCzTLCzPRPzPzCzTLCLFLPLOLTRORTLTLPzOLTLFRPzFRTLFLTRPzFzORPzFLCRFzOLCzFRFLFLFzPzORFzTRCzFLPLFzPLFLFRFRTRPzPRPRTLFLPRCRFRCLTLTRPLTLOLFLFLFRCROLORTROLTLPRTRFRFLOLFLCzTLCLPRCLFLFRFLCRFLPzCLCRPLTLTLFLFzTLORPRCLPzTRPRCzCRORFLCzPRCLTLTRPRPzPLTLPzFROLTLTRFRTRFRTLTLTRFRPLFLTRFRCLFzTRPRFLCzTRFLTLFLFzCLPRFLPLTROLPRTRFLCRPzCRFLTLTLCzPzTLFRTLCzORTRORCLTRTRFRPLTLCRFRORFROLFzTLFLFRFzOROLCzTRCRTLTRTLPLCRTLFRFzFRFzFLOLCzOLFLOLCLPzFRTRFLCRTLOLFRTLFRFzCLCzFLTRFLFLOROLTLTLORFRORFLCzFLFRFRORPLTRPRORTLTLCLORORTRCRFLFLTLFROLPLCLPRPLFRPzCLFLPROLTRFRFLTRPLFRTRCROLCRPzTRORFRPLORFLCLTRFRFzFLFROLCLFLFLFLFLCLORFRTRFLPRCRTRTLPLPRFLTLFRFLPLFzTLFRTRFLFRCzFLFzCzOLTRTLFRFRCzTLTRPLPLFLCRPLFLFRFRTLTRFLCRTRTRCzFLPzCRCLFRPRFRPRTLCRFzFRFLPRFRFRPRTLFRPLOLPROLPzFRTRFRFRPLOLPLFLCRTRPRFLFLPRPLPzFzPRFRCLPLFLFLFzPLCzPzFLCRCzFLTLTLTRFLCzFRFLTLPLTLFR
	3	FRFLTRTRFLTLTLORTRFzCLFRCzFLTLFzFLPLPzCLORTLTLPzFLTRFzCRORFLTRCRFRCzTLPRPRCzTLCLCzFRFLOLCzPzOLFRORPLFzCzFRFzTLFLFLFRPzOLCzTRCLCzPzFzPRPzCLPzFzFLFLTROLTRFRTRFRCRPzORTLFLFLFLFLFzCLTRFzTLFzTLTLFRTRCzCzPRTLPRFLPLCLFzFLFLTRFRFRTLCzTLFLFRCzFROLTLFLFRFLCRCzCLFLTLFzPLFLFLORPRFRPLORFLPRTRPRFRFRTRCRFRTRORTLCzPRTLFLFLORCzCzFLCzCRCzPLFRCLORTRFzORFLPRFzPzTROLOLORCLCRFRTLPzPRTLFLTLFLFLOLFRTRFLCzTLFzTLOLORPzTRCLPzTLOLFLTLTLFRFzORFzTLTRFRCzFRTLFLTRCRFRTLCzFRORTRPLPLTLFRORTLCLTLTROLFzFROLTROLFzTRTLPzFzOLFRTLORTLCRFLFLCRFRCzFLCRPROROLTLFRCLFRPzCzTLPzTLTRFLPzCzTRFLPzFRCLPRTRTLFLTLFLORPLTLTLFRCzTRFzCLORCLTRFLTRPROROLPLFLCLTLPRCLORFLFLCzFLPzFLORPLTRTRPRPLCzCLFLCLORFzFLFLFROLFRCRTLTLFLPRPLFzFRTLFRPRFLFLTLCRTLFLFRORTROLCRCLTLFRPzPLCRPROLPRFLPzFLCzFzOLCLTLFRFLFLFzFLFRFLOLCzFROLCzORPLPzTRFRFRFRFRTLCLTRORFRPRFRPLFzPLPRORFRPzTRTRFRFLFzCzFRTRFRORFRCzFLTRTLFRPLCLFLFLPLPzCRCRPLTRCRTLTLPzFLPRTLTRTLPLFRFRFRPLFLPzPLFLPRFLTLFRFLPLCLFRFLOLPRFLCzFRORTRFLFRFLCzOLTLTRTRPLFLPz
	4	TLFLTRTRCRFzORTLFLFRCzPRTRFLFzTLPLORPLFLCzFRFLORCLTLOLFLFLCzFzTRFzFRFRCLTLFROLPzFRCzTLORCRFRFRFLPzPRFLFzPzTLCLFRFzPzFLFLFRFLTRCLFLTLFLTLCzORPRTLCzFzTLFzPzCLORFzCRTRTRFLCLPzPRPzCzTRFLTLTRFRCzFLFzFLFRTLPRCzCRCLCRCzTROLTLCzFRFLORTRORPzPLFzCzPzOLFLCzCLFzCLFLFLTLFRFLTLFLFLCLCzCzFRFLCzPzCzPRFLFRCRTRTLFRTRTLPRPzFzPLCzPRTLFLFLFROLTRFRFLPRFRCzOROLTLOLPLFLOLTRTRFRTRPRTRCzPRORTRFLCzTLFRPRCRFLFzFRCLPRTLFRTLFzPzORFLORCLTRFRCzTLOLCzTLORFRORFRTRFzOLFzTLCzTLCLFRFLTRPLTLPzTLCRTLORFLTRTRORCLFRTLFLFRPzPLFRCLFzCRFLPLTRFRFRTLFLCzFLPLFLFRCRFzORPRCLORFLTLFRPzFRFzOLTLPLFzTLORPRPzFLTLPzPRTRCLTLOLTLTLTRPzTLFLCLFRTLTRORFLCRTLCLCzFRTRCzCLOLFROLTRTLORFLOLFRTLPLCLORFLFzOLTRFLPLORCLCRFLFRTRTLTLFRFLCzOLFLTRPRTRFRORTLTLOLPRFLORFLTRFLTRFRPRTLCRTRCLTLTRPLFRFLCzORCzFRTLCRPLFLOLFLPLFLFLFzPLORTLFLFzFRTRCRPzFLTLORFRCLFLPzFRFLPRFLPzCROLFRTLPLCRTLFL
3	1	CzPRPzORFRPzTLFRFROLTRFzOLORTLCLFLTLFLTLTRFRTLPzFzTLFzOLCRFzPzTLORTLCLPRCzTLFzORTLFLFzTLCLCzORFLTRTLTLFzTLPLFLFLOLFLCLTLFzFLPRTLTLFLFLFzFLFzFLFRPRPzCzPzFzTRORFRFLPzTLORTRORORPRCzFRTROLTLORPzCLORFzCLCROLPRFLORCLPLPzFzORTRTLCzFzPzCLFRTRPRFLTRFLTLPLFzTLOLCLORCzFRFRFLPLFLTLCLFLTRTLTRFRTLCLFzPzFRCzFLFLFLFLCLFRFRFRCRTLOLFRCzFLTRTLFLFzFRORCLFLORTRCRTLFRTLTLCRPRPRFLFLCRPzCzCRFRFLFLFLTRFRORPLPzTLPzFRCLORTLOLFRORFLFLPLTRORTLFRFRFzTLCzPzOLTLTLTRTLFRFLCLPzCzORCzCLCLTRFLTLCzFLTLTRFLFzTLFROLFRORCzFRFLCLFRTLPRCRTRPzPRTLCRFzCLTLFLPLFRFRTLOLFLFzFLFRCzTLFRTLFzCzTRFRFLCRFLOLTLPLORCRTLFLFRFRTRORTRPRFzPRTLFLPRFLOLFRTRTRFLORFRFRFLFRCzOROLCRCLFRTRFzFRPRFRFLCzPzTLFLCzTLFLFROLFRPRORPzFRCLPLTLTLPRTRCzFLCzTLCRTRFLPzFLCLFLPzFROLFzFLPzCRCzCzFzOLTLCzFLFLFLFLPRFROLFRPzCLTLTRTLFzCzTRPzFLCLFRFRFLTLTRFRORFRCzPLPRTRFLFRFLCLFRTLFLPRCRFRCRPLOLFRCzFROLCzORPLPzPLFLFLPROROLCRORFLFzFROLTRFzPLCLFLTRCRPRCzPRTRFzTRFLPzPLFLTRFRPzFLTRFLTRTRCLOLTRFLTRFLFLCRPLCLFRTLTLTRFRPRTLFLPLCRTLPLTRFLTRCLCzTLFLFRFLFLCLTRFRFR”
	2	FzTROLFRPRORPRCzTLFzFRTRORFLFRFLFLORFRCLFzTLFLTLTLFzFzFLORPzFLFRFRCzFLPRPLTLPRCLPzFRPzPRORFzFRORTLFLORTLPzFLTLORFzPzOLFzFLFzTRFLFLTLPRTRTLTRTLPzCRCLORCzTLCLFLFLTLFROLTLCLTLFzCRPzTLCLPzORTLORTRPzCzCRFROLCLCzCLFzCLTLFLFzFLFLOLOROLTLCzTLTLTRFLOLTLPROLCzTLCzTLFRTLTLTRCLORTLTLFRPLFzPLTRFLFLTLFROLFLPLTRORPzFzCLCzOLPzFRTLTRFRPLCLPRCRPRPzFRCRCLFRTRTLFLFzFRFLORCLPRTLPzCLFzPROLTRFLFLPzTLTLPRCzPzCLFzTLFRTLFLTRCRFzORTRCLOLFRFLFLTRFzFLCLPzPLFRFzTRTLCLTLPRFLFRTLFLFRCzFRORPLFRPzORFRFLCzOLORTRFRTLORFLCzCLFRCzCRCzFRTRFLFLTLFRTLCzFLTLFLPRFLTLPzFLTRCRTLORFLPRFRFLORCLFLFRORFzCzTLPRFRTLFROLFLCRCRFLFRCzPzTRFLFRPLCzORTRTRFRFLFLTRCzPzORFLFRCzORFzTLCLCRTRORTLCROLFRFLFRFzOLFLTRFzFRFRFRFRORCRTRFRPRCRTLCzFRFRFLOLFLFzFLCLFLFRFRFRPzTLFRPLCRFLTRPLFLTLFLFLPLTLCLCLFLCzFLFRFRTLPLFLCzTLOLPLORTRCRTLOLPzFLPLPzFRCLFLCLFLOLFRCRFRTRFRFzFLPzFLTRCzTRPzFRFRTRCzFLCLPRCzFzORFRFRCzTRPRPLFzTRCLFROLCzOLCRTRFLFLCROLPLTRCRCzTLCzTROLTLFRFLPzOLFLFLPzPLTLFLTRPzFLFLTLCRPzFLORTRPRFLTRTLTRTLFRCLFR
	3	PRCzOLFRFzTRPzFRFRTRFLORTLORFzCRFRTLTLFLFLFzFzTLORPzTLCLTRFLTLFzPzFLTLCzFLTLPRCLFzFLTRFzCLPzORFLFLFLTRORCLTLORTLFLTLFRFLFRCzCRFRPzCzTLFzTLTRCzCLFRORFzFLTRFLFRPzORTRFzPLPRORPROLOLORTLFLFzORTLTROLTLORTLOLFzTLFRCzCRTRTLTRFLTLPzOROLCRCLFzPzFLPLTLFLPzPRFRCRCzFRTLPzOLTLFLFLCzORFRFzPzTLCRTLTLTLORFLFRCLPRPzFLPRFLOLFLTLTROLOLTRFLCzTLFLCzPzTLPRORFzTLORPzFzFLFRCLCzFLCzFLFLPRTRFRPzFRPLFRFRTRTLCzTLPzORCLFLFLTRFROLTLPROLCLCzFLFzCLPzTLPLCLOLPzFRTLORCLORTRPLPzFzFLORPLPRCRFLFRCRFLTLTLTRFRFzOLFLTLTRFLCzTLORFRCLFRFROLPRTLFLFzFLFLTRCLFRCzFLCRTLFRORFRFRTLFLFRTLPLORCzFRTLCLORFRTRPzCLCLTLCLCRFLFRPzFLFRFLFRTLFLORPRFLCLFRFRCRCLFzFLTRTRFROLFLFLFRTRCRCzTROLFRTLOLFRORTRCzCRTLFLTRFRTRFRORTRFRFRFLFzPRCLFLFLORTLCROLFRCzFRCzFzFRPRFzCzCzFLTRORCRFLCRTRFRFLFLPLPRFLTROLFLOLFRCLPzOLTLPLTLCLFLCLCzFRCLFLCzFLFLTLTLFLPzTRPLORFzFRCRTRPLFLFLPzFLCLFzFRFLFzFLTRFLTLFROLPLCzCLFRPLFzCLPLFzTLTRTRFLTRTRTLTLTRORPRFLCzTLFRFRTRTRTRTLOLOLFLFRFzPzPLCLPRTLFRFLFRFLTRPLCRFRFRTRPRCRCzCLTRCRFzCRFRFLPzFLOLCROLFLFLPzFRTLCzCzOLOLPLTRCLPLTRCLTRPLTRPLTLPLTLFLFRPRFRFRPRFLCRFLPRCRPzTRFRTLTRPzFLFRCzFLPRFLPzCRFLPRFRFRFRCzTROLTRCzPLORFLFRFRCzPRTRTLTRCRTRPzTRPLCRFRCRPROLFLPRTLFRTLPLFLOLPROLCzCRORPLPRFLFLTLFLTRFRPzTRFRTRPLFRFRFRFRPRPRPLFRFLPRFRFRFzFRFLTRFRTRFzTRFLFRFLTRPLTLCRTRPRTRCzFRPzTRPzPLOLOLCRFLFRFLFR
	4	TRFzCzPRFROLFzTLFLTLTRFRCLPRCLTLCRFzOLPRFLFRFzOLFLFLORPRPzORFLFzCzTLCLFRFRFLPLFzFRPzTLFLFLTRFzFLCzPzORFRFzTLTLFRPRFLTLORTRTLCzFRPzTLPRPzPzPRFLCLTLCLTLCRTLFLOLFLTLOLCLCRTLCzFLPzTLPzPLFLPRTLFRTLCzFLFRCzORFLOROLFRFLFLTRTLFzFRCRTRTLPzFzFLTLFzPzTLTRORCLFLORTRCRPzCLPzOLTLFRCLOLFRCzTLORORTLTLFLFzFRCzCLPzCzCRCzFLFLFLFLFLPLCLTRTRPRPLFRPLPzCLCzTRORORTRCRTLTLCzCLFzTLPzTLTRPzFRPLORFzOROLORPzTLCRFRFLTRTLFRPRTLFRTRTLPRTLOLCzFRFRCLCRCLTLPLCRTRCLOROLFzFRORCzFLCLPzPRFzTRORFLFzTLTLOLFRFRTRFLTRFRCLORPzOLFRFLORFRPRORTLORFzFzFLCLPLFLCRTROLFLFRFLFzCzORTLCLFROLFRTLFRFLCRPRCLFLTLFzORFLTLTLTRFLFLTLFLOLTRPzFLTLTLCRFLPRCzFzCzPLFLPRFLCLTLFRFRCzPzFRFLCRORFRFLPzPzPLFRTROLTRFRFLFLCzPRORFLFLFRFRPRCzFLOLORFLPLFLCLFRFzTRFRTLTLFLCLTLTLPRPLTLFLCLCRPLOLFLFLFRTLFRTRCzTRTLORCzOLPzPLCLFRFRFLCzTRFRFzCLCRFROLTLFROLPRFRORTROLFRTRFRCRFLOLTRFzCzORFRTRPLCzFRCzTRPRTROLTLFzFROLCzFRFzFLCzFLFRPLTLCLTRCzFLTRCRFLTLCRFLFRORFLPzFLFLPRTRTRFLPzCRFLFLTRFLTRTRFzFRCRTRTRFLPzFLTRPLPROLTRFLFRPRFLCRFRPzPLOLPzFLFzCRFRCROLCRPRCLTRPzTLTRFzPLFRFRTRTRFRCRFRTRORCRPLCRTRFLTLPLPRFRFRTRFLOLCLFLFLTLOLPLFRFRCzTLOLFLFRTLPRCRFRCLTRCLTROLPRFLPLTRFRTLFRFzFRFLCRFRPzFLTLFRTRTLTLTRTLFLTLFLPzTRCRORCLFRFRFRTRFzTRCLOLCLCzOLFRFLFRFLTRFRFRPLFLORPzFRPRPLFLTRCRFLFLFRFLPRFRPRCRPzFRFRFzFLCRTLORPLFLFRFRTLPzTRPLFRPRFRFRPLFLFRCzCRTRFRPLFLTRPRFRPzFLPLFRFLPLPRPLCzPRFLPLTRCLFLFzTRTRCzFRPL
4	1	FRTLTRTRFLFzFRCLORTRFLFRCzFRTLCzFLFLFRCzFRCzOLFLTRTLPzTLCRCLOLORORFLTRFLTLTRPRTLTLCLPRORPLORTRORFRTRFRFLCLPzFRFLFLTRPLFLOLTLCLFRCzPRTROLTLFzCzORCLCzCRFRFLCzCzCLTLFRFLTLCLTLTLCzCzPzTRFRTLOLPRPzCLPRCRFzORPzTRFROROLORTLORFRORFRFROLPzCzFRCROLPzFzFRFRFLFRFLTRFRFzPzPzCLPRCLFzOLCRTRORFzOLTRFRPRTLCRPLCzFzTLFLFLTLPRPLFLTRCLTLPLFzORORCLFzCLFLTLFLORTLFRTLCLPzPRPRTLOLFLPRCzCzFzFLCLFLCRFzFRCRFLOLFzFLPRTLFLCzTRPzFRFLFRCRFLFLTRFRCRCzTLPRTRCRTRCzFRTLORTLPLTLPzCzTLCLFLFRCzFRFLTRFzCzFLTLTRORFLCzPLCRPzCLTLTLFzFRORCLORTLFRCzFRPROLPzORFROLOLFRCLFRTRPRFLFLFzTLORCzPzFzCLFzTLORFzFLTLFLTRFLCRFLFLFLPRFLTRCzFRORFRORFLFLCLOLOLFRFRPLFLORPRFRTLFLPRFzCRCzFzTRPRPLFRPzFRTRTLOLTLPzPLPzPLTRCzOLFzPL
	2	FzFLCLCRTRORFRFLCLFRFRCRTLOLTLPRCzFRTRTRPzPRORPLTLTRTLFRPzCzFLTRFLFRFRCzORPRPzCLFLOLCzFRFRCLTLPzCLCzFRORCzTLTRCzFRCzORCzFRTRFzCROROLFLORCzCLPRFRCLFzFRFzFLFLFLFRCLTLTRPRORTRFLFRPRCzCRFRTRTLPzOLFRTLCzPzTLORORFzFROLTLFLFLPLFRFzFzORFRTRFRORFRCLTLFLOLTRPzTLCzFLFzTLFLCLCLTLPRCLTLCzFRTRCzPROLORFzOLPRPzTRCROLTLORFLCLORTLCLPzFzFLTLTRFRPRTRFRPzCzTRPLTLFLPLFRTLCLTRTLPRFLFRCLFLCRTLFLCRCzORCLPROLFRFLFLCzTLPRFLFLCzCLPzORFRPRTLTROLTLFzFROLPLPRPLCzCzCRFLFRFROLCzPLFzCzORPzORTRPzCROLPRCLFLPLTLPzORTRFLTLCLFLOLFzTROLFzCzFRTLPzTLFzPLFzPzTLFLTLPzPLTRFRFLTRFRTLTRFLOLCzTRTLFzTLFzPzPLFRTRPRTRCRORFLFRFRORFROLCLFRPzCRFRFRFLTRFzCzFLFLFzTLCzFzCLPzTRFLCLFRCzORFRFLPRFLCzFLTLFLCRFRPRPLFLORCLPROLOLFRCRFLFRPLPLCRPLTRFzFLTRPLPzFRORTLTRFLFzFRORTLFLORTLTLPRORCLTRFzPRCLFRTLFRFLFLCzTRFLPzFLFLOLCRFRFRTROLPLOLFLFLOLPLOLTLPRTLFLCRFRPLOLFRPLPzFLFLTRCzFRFRFLFzTLORTRTLTLFLFLPzCzFL
	3	TLCLTLFRFLOLOLFzTRTRCLCRPzPRTLPzFzFLCLCzCzCLCzFRPRCzCLFRTRORTLPRFRCzFRCLCzPzPRPzOLFRORPLPzOLCzTLFzCROLFROLORPzCLTLOLORFzPzCzFRCRCLPRCzTLTRTLTRFRFLPLFRFzFLFRFRORCLTRCzTRCLPzFLTLFLFRTROLCRFLTLORPRTRTLTRFLFzTRCzFRORTLFLFLFLCzFLCRFRPzORFRTRFzOLTLCzPRFRCzPRPRCLOROLFRPzTRPLORFLFzTLFLTRFRFLFRTLCLTLFLFRCRTLORPROLTLFLCzCLFLPLCzFLFLFRFzTLPRPLPzFRTLFRTRFzTRFzCLCRFRPzTRTROLFRTLFzPzFROLFLPLCzPLORTRFLCLCLORTRFLTLFLCzFRTLFzPLFRFRTRPzFzFLCRFRCzFRCzPRTLPRFLFLOLOLTRPLTLCLFzCzFLTRPRPLFzFzORORFLTLCLTRCRFROLCzTRTLFzTRFLFLFLTRFzFRFLCzFRFRCLORPzCzCRFLORFzCzTRCRFLCRPzTLFLTLORFLOLCLORCzFLFLTRPRPzTLTLCzFLFRCzFLFLCRFzPLPLOLCLTLFLTRTLTRORCLOLFLFLOROLPRPzCRORFROLTLORTLFRPzFzPRPLTLPzFRFLFLFLPRCzORTLTLORFRTRTLPRTLPROLCROLTRTLFLFzFLTRPLCRFRPLFLCLORTRTLFLORFRFLPzFLTLPRORFzFLOLPLTRCzFLTRPLPROLPzFRFRPLORFLFRPLFLFzFLCLTRTLORTLFLFRCRFRFRTLFRFRFRFzFRPRPLFLFLFRTLTLFROLCzFRPLCzOLPLTRPRFRTRFLPRFL
	4	FRCzTRTLFRTLTRTRTLCzCzPRCzCLFRCLFRFLCzFRTLTRFROLOLFLOROLCRCLCLPRCRFRCLFRCzORPLORFzORCLTLOLTLOLPzTRFRFLFzPzTLCLPzPzPROLFLORPzORFRTRFLFRFLTLCzFLCLFLORPRPzTLFzPRCzTLOLPRORFRFLPLFLFLFLPzFRFLFLFRTRCzFRORTRFRORCRFzPRTLCLCzOLTRTLFRPzCLTLCLFLORCLFRCLFLOLFRFROLPRCLTRFRFLTLFLTRTLPLFzFRTLPRFRCzORFzTLFRPzCzORFRFLFRTLPzCzPzPLCzTRFRFLCRFRFLCzPLTRCLCRTRCRFLOLFzCzTRFRCRTROLTRCzFzTLPzPLFzFRCRFLFzOLOLORFzTRPRTRCzFLFzPzPRTLOLTRCLFzFLTLFLFLFLCzCzFRTLORPLTRORCLTRORCRFLFLOLORCzTLFLFLTRFLCzCRCzFLFRFRPLPLFLFzCLCzTLTLFRPzFRTLPzFRPRTRPRTRFLPROLTLTLTLCRFzTLPRPLORTLTLFLFLORPRTRTLORCzTLOLPRPRFLFRORORPRCzFzPzFzPzORFzFLCLORFLPRTLCLTRFzCzOLCLOLFRCzTLPLFLTLTRCLFRORFRTRORFLCLTLOLPLFRCLFLCzTLTRFRFRFLTRFRCRTLOLOLFRTLFzTLFLFRFzFLFLTRORFzPLFRFLTRPzFLFzCROLCzTRPLTRORFLPzOLFRTRFRPzOLCRPLFRFLTRPRFRTLTRFRFRPLFzPRFzFRTLPLOLTRCzFLFRFLPLCRFRFRTLFLPRORPLTRPLCRPLCLFLFLTRFLCLFRTRFRFRTLFRPzTRCzFLFRFRFzFLFzCzCRTLPzTLPRFLPLCLCRPzPLFRTRFLOLCzFROLPzFLPLPRORFLTLPRFRPRFRFLPRFRORFRFLTLFLFRTLCRCLCRPzCRFLTLORTRFLPLCRTRFRPzTRFLFLFRFzFLFLPLORTLTLFRTLFLPLPzFRPRTRCRCRPRTLFLCzFLTRFLTRFzFRCRTROLCLTRPRFRFLFLFLFLFLOLTRTR
5	1	FzTLFLCzTRFzFLCLORFLFLORPLFzFLTLFLORTRTLCzTLFLTLCRFzORTLFzTRFLFLPzCLTRFRPRFLTLFLFLFLTLFLFRFLFRPzCzPROLFLFLFRTRFLTLFzCzFLFzPzFLFzPRPzTLTLFLTLTLPzPzCRTLTLTLPRFRTRTRCRPRTLPzORFzFLFRCRFLPzTLFzOLPzCzFRTLCLPLCRCzPzCLPzFRTLPLTRFROLTLFRTRFRFLFLPzFzTLCRTLTRCLCRTRORFRCzTLCLTRFLTRPLCzCLCLTRCLPRTLORPRCzCzFRFLFzFLTLPzTLFzOLFRFRPzCzORFzTLFLTLCzPzPRTLCRCRTRFRTLTLPzPLPzCLTLCLORFLFLFzFRPLCzFLPzTRPzFRCRTRTLTRFLPLFRPLFLCLTRFzORFRFLPLFLTLOLFzCzFzTLTLORPLORTLFLPzTLCzFLTRPRTRCLTRCzCLCzFRCzTROLFLPLPRTLCLPLTRCROLFRCzOLFRFLTRTLCzFLCzFzTLFRTLFRFLCRTLFRFLCzPLFLCRCzTRFROLPRFLTRCLFRTLOLFRPzFzCLFRFLFzPLTLPLFLFLFLTRCLFzFzCRFLTRCRPRPLPROLTLCLFRTLFLPzFLTRFLPLTLPzFRPzFRTRPzCLOLOLCLFRFLFLPLPzOLPLTRPRTRFRCLFRTLTLTRFRORORFRORCzPLFRORTRFRFzFzTRFRTRFRPRFLCLFzFLTRFLCRFLORFLFLPRORFRFRFzPRFLCzTRCLFLFROLFLOLPzFLORCLFLTRTRPLPRTROLFzCRFLFRPRCLFRFLOLCRCLCzORPRORFLCzFRCLFRORCzTLCRTLFLPLTRCzTRCLPRFLOLFLCLFL
	2	PzTLORFLFzCRTLFzFLFLFzPLTRFzFLTLFRTLPzFRTLFzFLFRTLORTLTRCzFLCLCzTLFLTLCzFzTRPzFLCLFLFLFzFLTRPzFzFRTRFLFLFLPRFRCzFLTRFRFLPzFLFzTLPzPRFLCzPzCLFzPzTLFLTLPRFLOLFLORFzTLORFLORPzOLFROLTRFLFLTLFLCRPzPzCzPRTLFzFLCzTLTRFRTLPzCLTLCLPzFLCzTRTLFzFLTRCLCLTLPRCzCRFRPzOLORTLCzFzPzFRTLTLFRCzTLFRTRFLORTLTLTLFRPLCLFzCRFRCRTLORTRCzPzFLTLORFzPzPLFLPLTLCLTRCRCzPRPRTRTLFRPLTRPzTROLFzTLTROLTLPzTRCzFRFzORTLFLPLFLCRCLFLPLFLFLPRPzORCLCRTLTLFRPLFzCzORFLFRPRORCLTRFLTLTLPRFRTLTRFLPLCLPzTLFRPzCzFLTRPLPLORFRCRTRPLCzCLCzFRFLTRFRTLFROLCzPLFRFzTRCzFzFRTLOLTRCLCLFLCRTRFLPzOLCzTRPRCRFLPRCLCRPLFRTRPLFRFRCLCzFLFzFRFRFRFRTRPLTLPLFLTLTRFLTLCzTRFLORFLFRFLFROLPzPROLOLFRCLTRCRTRCLORTLPzOLCLOLTLFRORFLFRFRORCLFRTRFLFLTRCLFLFzCzORFRFLTLFLFLTLFRPRCRFLPRFLCLCRFzCzCRTRTLPRFLORPLTLTRFLCzTLPLFLFRTRPRFLPLFzOLCzTRPzORCLCRPLFLFRTLFRORTRFzTRTLTRFLTRFRORFRFzFL
	3	ORFLTLFzORFLFzPLFLTLFzCRTLORFLCzFLOLPzFzTLCzTLFLPzPRPzFRTLFLPzCLCRPzCLFLTLTRPzFLCLCzFLFLFzTRCzFLFRFLPzOLPzTLFLTRFLPRORTLORTLFLTRFRCzTRFzFRPLTLFLCLPzFLFzTLFzTLFLFLFRPzFLCLTLFzTLTLFRCRTLCzFRFRTRFzPzFzTLCLTLFLFLCRFRTLTLCzFLORPzCRFRTLCLPLFLOLTLTRCLTLPRTRFLPRTLFRTRPzCzFRTLCzPzTRTLPRCzFzFLFLTRCzFzTLPzCRTRFzFLCRPLPRTRFLFLCLFzFzORCzCLTRFRPzPLFLFLOLFzCRCLPzTRORCLFLPLCzPRCzPLTLTLTLPRFRTLFRTRFLTLFLCzCRTLFLORFLPLTLFRPLFzTLTRTLPzCLTRCRTRFLORPRCLTRCRFzCLPzTROLFRFLTLFRTLCLFLORTRCzFLORFzPzORPzTLFLFRPLTRFLFLPLFLFLFRFLPzPLORFLFRFRTLFROLCRFLPRPzFRFzFRCzPLPzCzFRPLTLTLTRFRFRFzFRCzTLTRCLTRCzTLOLFRCLFLPLTRPLCzOROLTRTLFRTLPLFRCLFRCLFRPzPLCLOLTLCLCRPROROLFLFLFzFLTLCLTRFRORFRPzFLFRCzTRTRTLPLFRFLTRCLFRFzCzTRFLCzCRFLTLCLFLTRFLTROLTRPRFzOLFLPRTRFLORPLCLORCRFzFRORFLCLORPRPzFRTRCRFzFRORFRPRFLFRPLFLPzTRORPRFRTRFzFLFRFLFRFLCRCzTRTLCzOLPzPROLTLFRPLFLTRPLORFROROLFRFzFLCLFLTRFLPRFLOLOLFRTRFRTLFLOLFRFRPRFRORTLTRFRFzCzTLTRORTRTLOLCRFLCRFRCRCRPRCROLOLCzPRPLPLTRFRPRFRTRTLFRCzOLPLCRORFRCRORTRFRFLCzFRTRCLOLTRFLOLORPRCRTRFRFRCzPRFRCLFRFRCRTRFRFRCRCRFRTRCzORCzFzFLOLCLFROLCRCzTRTRTRPRFRPRFRFRPLPRFLTL
	4	PLFzFzPzFzTLFLCLFLORTLFLTRFLCRFzFRPzORTLFzTRTRFzFLTLPRPzFLCzFLORTRTLFLFLPzCLTLFzFzFLTRFRFLTLCzFLPzFzFLTLOLFLFLFRFLFLPLFRTLTLTLFzTLFRCzCLTLCLCzTLCzPRPzORTLFLPRFLFRTLPRTLFRCzTRPRFzORFRFLCLTLPzTLFLPzTLFRFzOLTLPRCzFRTRFLTRCRTLCLFLFzPLCzTLTLFLFLFzTLTLTRFRTRCLPzCLTRCLORPLPzCLPRTLCzCzFLTLORTRTLTLORTRPLPzTLFLTLOLPzFLPzORFLTLPzTRPzPzCRTLCRFROLPRCzFRCRFRCRFzCzFLFzFRFLTLOLCLCRPLFLFRFLCzFRORFzTRCLTLCRFRTLPzCzPzFRFRFLPRTLFLPLORFLFzPLCRTRFRPzPLFLCzPzTRFRFRCzPLCRFLFLTLFLOLFRTRORORPLTRTLFzCLORPRPzPRFLTRTLFRTLPzFzFRTLOLCzTROLPRCzFzPLTRTRFLORCzCLCzTLFRCLPLFLTRTRFRTLFLTROLCzFLPLFLFLTRTLPLCLTRFLPLFLCzFRFzTLFLCLFRFLFLFRFzFRFLFRFRCzOLFLCzPLTRTRFLTRPzFRCLCLOLPzFLCLFzORPzTRCzCLFRFLCRCLFLCLFROLTLTRCRPLORFRFLORFRPRTRPzOLORFRTRCzPLTLFRORFRTLCRPRORTRCRFLFRPLPzPLPLPRCRCzFzCROLFzTRCLFzFLFLCLFLTLPRTRPzFRFLTRCRTRFROLFRORCLFLFRPRCLFLCRFLPLFRPRFLFRPRFROLFRCLTLTRTRTLFRTRORTRPRFLOLFLOLCLORFR

Histograms of symbols used in DLob sentences are shown in Figure A1.

## Appendix B

**Algorithm A1.** Training and evaluation of the SOXFE model under nested participant-grouped cross-validation
Input: EEG segments X (19 channels × 7500 samples), labels y, participant identifiers p. Output: Predicted labels of all segments and participants, performance metrics. 1: for each segment: compute TS1 and TS2 with BCT (Equations (1)–(4)); compute the transition tables tt1–tt4 with PNPat (Equations (5)–(9)); normalize them (Equation (10)) and vectorize them into F1–F4 (Equation (11)). // label-free, applied per segment 2: partition the participants into 10 outer folds so that all segments of a participant belong to one fold. 3: for each outer fold k = 1, …, 10 do 4: Train ← segments of participants not in fold k; Test ← segments of participants in fold k. 5: for each feature vector g = 1, …, 4 do 6: compute min–max parameters on Train only and apply them to Train and Test (Equation (13)). // fitted once on Train; not refit within inner splits 7: CWINCA on Train: NCA weights and ranked indices qin (Equation (14)); cumulative weights cw (Equation (15)); start = first i with cw(i) > 0.85; stop = last i with cw(i) ≤ 0.9999 (Equations (16) and (17)). // fitted once on Train; not refit within inner splits 8: for q = start, …, stop: loss(q) ← participant-grouped inner-validation error of kNN with the q top-ranked features of Train (Equations (18)–(20)). 9: q* ← argmin loss; sind_g ← qin(1:q*) (Equations (21)–(23)). 10: tkNN on Train: inner-validation predictions for 30 parameter settings (3 distances × 2 weights × k = 1, …, 5; Equations (30)–(34)); IMV over the top-l settings, l = 3, …, 30 (Equations (35)–(37)); select the best of the 58 outcomes by inner-validation accuracy (Equations (38)–(40)). 11: end for 12: CMV: inner-validation accuracy of the four classifier-based outcomes and of the four voted combinations (Equations (41)–(44)); select the final configuration greedily (Equations (45)–(48)). 13: refit the selected kNN models on Train with sind_g, predict the Test segments, and apply the selected CMV combination. 14: participant-level prediction ← majority vote of the segment predictions of each Test participant. 15: end for 16: compute the metrics from the pooled outer-fold predictions; Test labels are used only in this step.

Algorithm A1 describes the implemented participant-grouped procedure. Steps 6 and 7 are fitted once on each outer training set and are shared by its inner splits. Complete nesting would repeat both steps within each inner training split before Steps 8–12; this variant was not run.
